# Endometrial organoids: a reservoir of functional mitochondria for uterine repair

**DOI:** 10.7150/thno.90538

**Published:** 2024-01-01

**Authors:** Sun-Young Hwang, Danbi Lee, Gaeun Lee, Jungho Ahn, Yu-Gyeong Lee, Hwa Seon Koo, Youn-Jung Kang

**Affiliations:** 1 Department of Biomedical Science, School of Life Science, CHA University; 335 Pangyo-ro, Bundang-gu, Seongnam-si, Gyeonggi-do, South Korea.; 2 Department of Biochemistry, Research Institute for Basic Medical Science, School of Medicine, CHA University; 335 Pangyo-ro, Bundang-gu, Seongnam-si, Gyeonggi-do, South Korea.; 3 CHA Fertility Center Bundang; 59, Yatap-ro, Bundang-gu, Seongnam-si, Gyeonggi-do, South Korea.

**Keywords:** Asherman's syndrome, Endometrial organoid, Mitochondria, Infertility, Uterine repair

## Abstract

**Background:** Asherman's syndrome (AS) is a dreadful gynecological disorder of the uterus characterized by intrauterine adhesion with severe fibrotic lesions, resulting in a damaged basalis layer with infertility. Despite extensive research on overcoming AS, evidence-based effective and reproducible treatments to improve the structural and functional morphology of the AS endometrium have not been established.

**Methods:** Endometrial organoids generated from human or mouse endometrial tissues were transplanted into the uterine cavity of a murine model of AS to evaluate their transplantable feasibility to improve the AS uterine environment. The successful engraftment of organoid was confirmed by detection of human mitochondria and cytosol (for human endometrial organoid) or enhanced green fluorescent protein signals (for mouse endometrial organoid) in the recipient endometrium. The therapeutic effects mediated by organoid transplantation were examined by the measurements of fibrotic lesions, endometrial receptivity and angiogenesis, and fertility assessment by recording the number of implantation sites and weighing the fetuses and placenta. To explore the cellular and molecular mechanisms underlying the recovery of AS endometrium, we evaluated the status of mitochondrial movement and biogenetics in organoid transplanted endometrium.

**Results:** Successfully engrafted endometrial organoids with similar morphological and molecular features to the parental tissues dramatically repaired the AS-induced damaged endometrium, significantly reducing fibrotic lesions and increasing fertility outcomes in mice. Moreover, dysfunctional mitochondria in damaged tissues, which we propose might be a key cellular feature of the AS endometrium, was fully recovered by functional mitochondria transferred from engrafted endometrial organoids. Endometrial organoid-originating mitochondria restored excessive collagen accumulation in fibrotic lesions and shifted uterine metabolic environment to levels observed in the normal endometrium.

**Conclusions:** Our findings suggest that endometrial organoid-originating mitochondria might be key players to mediate uterine repair resulting in fertility enhancement by recovering abrogated metabolic circumstance of the endometrium with AS. Further studies addressing the clinical applicability of endometrial organoids may aid in identifying new therapeutic strategies for infertility in patients with AS.

## Introduction

Asherman's syndrome (AS) is a rare and dreadful gynecological disorder of the uterus, which is a consequence of trauma to the endometrium, especially the basalis layer, where endometrial progenitor cells are localized. Severe trauma after surgical procedures, primarily dilatation and curettage (D&C) or viral infection disturbs the periodic regeneration of the functional layer of the uterus, resulting in menstrual disturbances, such as amenorrhea or hypomenorrhea 1-3. Key features of the endometrium with AS include intrauterine adhesion of the uterine wall, which decreases the volume of the uterine cavity 4, 5. In addition, severe fibrotic lesions triggered by abnormal tissue repair under chronic inflammation induce poor epithelial growth and vascular development and the extracellular matrix (ECM) displacement by excessive fibrous connective tissues, strongly leading to dysfunctional reproductive systems 5. Many retrospective studies have shown that patients with AS suffer from severely impaired fertility such as repeated implantation failure (RIF), recurrent pregnancy loss (RPL), or intrauterine growth retardation (IUGR) 6, 7. Therapeutic strategies adopted in clinical trials include hysteroscopic adhesion lysis, intrauterine balloon stent/Foley catheter to prevent re-adhesion, and postoperative adjuvant hormone therapies. However, their therapeutic efficacy is inconspicuous due to high recurrence rates of intrauterine adhesions 8-12. Recently, stem cells from diverse sources such as menstrual blood, umbilical cord, and bone marrow have shown exciting potentials for the uterine repair and endometrial regeneration in animal models 13-15. However, detailed cellular and molecular mechanisms of anti-fibrosis underlying uterine repair are still required to elucidate for allowance of the evidence-based clinical transition.

Chronic inflammatory signals including nuclear factor kappa-light-chain-enhancer of activated B cell (NF-kB) and transforming growth factor β (TGF-β) signaling pathways invariably generate fibrotic lesions due to a dysregulated fibro-proliferative response and abnormal tissue repair mechanism ultimately resulting in irregular tissue construction and function 16-18. Fibrotic lesions are mainly characterized by an excessive deposition of the ECM and inhibition of its degradation and remodeling, predominantly expressing fibrillar collagens (collagen types I and III) 18. This is due to aberrantly metabolically active myofibroblast cells accompanying with increased expression of α-smooth muscle actin (α-SMA) and tissue inhibitors of metalloproteinases (TIMPs) 4, 19, 20. Furthermore, in fibrotic lesions accumulated oxidative stress and excessive reactive oxygen species (ROS) cause damages in endometrial cell-matrix, protein, and DNA, leading to the aberrant physiological function of endometrial tissues.

Unlike traditional two-dimensional cultured stem cells, organoids can preserve the key features of the native tissue and maintain self-renewal capacity and cellular diversity for a long-term culture in the three-dimensional *in vitro* culture condition 21. Various tissue-specific organoids, including the heart, brain, small intestine, kidney, and endometrium have been introduced and have been widely utilized as a platform for drug screening and a potential cell therapy tools for tissue repair 22-26. Recently, endometrial tissue derived-organoids have been generated and shown long-term expansion with epithelial cell-enriched characteristics, recapitulating the physiological functions of the human endometrium responding to sex hormones 27-29. However, little is known about its potential as a regenerative medicine for damaged or diseased endometrium. In this study, we successfully generated mature endometrial organoids and established a murine model of AS that displayed a structurally and functionally compromised endometrium resulting in significantly delayed and suppressed fertility ability. Moreover, we firstly provided the experimental evidence of the feasibility of endometrial organoid-based regenerative therapeutics for the endometrium with AS by uncovering the underlying cellular mechanisms.

## Materials & Methods

### Human samples

Human endometrial tissues from normal or AS patients were obtained from the CHA Fertility Center Bundang after diagnostic hysteroscopy. Samples were obtained for research purposes with informed consent of the patients. For histological analysis, 1 normal and 1 AS endometrial biopsy were used, and 3 endometrial samples from healthy women were used for three independent sets of organoid generation for the purpose of organoid transplantation in AS endometrium in BALB/c-nude mice. This study was allowed by the Institutional Review Board (IRB, approval number 2020-10-007) of the CHA Bundang Medical Center.

### Animal uses

All animal procedures followed animal care guidelines approved by the CHA University Institutional Animal Care and Use Committee (IACUC, approval number 200193, 210055, 220011, LMO approval number LMI 22-852). C57BL/6 female mice (6- to 8-week-old), male mice (7- to 9-week-old), BALB/c-nude female mice (7-week-old), male mice (7-week-old) were purchased from Orientbio (South Korea). C57BL/6-Tg (CAG-EGFP strain #:003291) mice (male, 7-week-old) were provided by the Jackson Laboratory (USA). Genotype analyses were conducted via tail snips using gDNA Extraction Kit (Bioneer, South Korea) for maintaining lines. Mice were housed under standard temperature and humidity with light-controlled (12 h light/12 h dark) conditions in SPF environment.

### Establishment of a murine model with Asherman's syndrome

The female mice were anesthetized via intraperitoneal injection of 2,2,2-Tribromoethanol (Avertin) and used for AS modelling as previously described with small modifications 25, 30, 31. Briefly, the needle tip of the 26 G 1/2 syringe was bent by 2 mm at an angle of 45 °C and inserted into the uterine horn. The endometrium lining was scratched 20 times in four directions when uterine wall became rough and bloody, but the perimetrium remained intact. Mice were sacrificed at 2, 7, 14 and 21 days after surgery and uteri were harvested for model validation.

### Generation of human endometrial organoids

Endometrial tissues were minced into 1 cm^3^ and dissociated into single-cell suspensions by incubation with 50 ug/ml Liberase (Roche, Swiss) in HBSS (Gibco, USA) at 37 °C for 30 min. The mixture was passed through micro-pored strainers with different sizes (SPL, South Korea) to obtain epithelial-enriched fraction as previously described 27. For 3D culture, cells were suspended in DMEM/F12, mixed with 6 mg/ml TeloCol - 6 (Advanced BioMatrix, USA) and added neutralization Solution (Advanced BioMatrix, USA) at ratio of 1:1 (v/v), finally seeded in 48-well culture plates. After solidification, the droplets were then maintained with organoids culture medium consisted of DMEM/F12 (Gibco, USA), 1 X N2 supplement (Gibco, USA), 1 X B27 supplement (Thermo, USA), 1 % P/S (Invitrogen, USA), 1.25 mM N-Acetyl-L-cysteine (Sigma, USA), 1 X L-glutamine (Gibco, USA), 10 µM Y27632 (Biogems, USA), 10 µM A83-01 (Peprotech, USA), 1 mM Nicotinamide (Biogems, USA), 50 ng/mL recombinant human EGF (Peprotech, USA), 100 ng/mL recombinant human Noggin (Peprotech, USA), 500 ng/mL recombinant human R-spondin1 (Peprotech, USA), 100 ng/mL recombinant human FGF-10 (Peprotech, USA), 50 ng/mL recombinant human HGF1 (Peprotech, USA), and grown at 37 °C under 5 % CO_2_ as previously described 27, 29. The culture medium was changed every 2 days and passaged every 6-7 days.

### Generation of mouse endometrial organoids

Mouse endometrial tissues were mechanically and enzymatically digested with TrypLE Express (Gibco, USA) for 10 min and enzyme solution involving 1.25 IU/ml Dispase II (Sigma, USA) and 0.4 mg/ml Collagenase V (Sigma, USA) for 1 h at 37 ˚C. After incubation, solution was filtered through cell strainers to remove the stromal fraction, the cell strainers were washed with a fresh PBS to collect epithelial fraction. Isolated epithelial fraction was suspended in DMEM/F12 medium, mixed with Matrigel (Corning, USA) at a ratio of 1:10 (v/v) and gently seeded in 48-well culture plates. Cells were then covered with organoids culture medium and grown at 37 °C under 5 % CO_2_.

### Cell culture

Mouse endometrial stromal cells (mESC) were obtained from dissociated stromal fraction. Filtrate were for 5 min at 1500 rpm for 5 min. After removal of supernatant, the pellet was resuspended in DMEM/F12 medium supplemented with 20 % FBS (Gibco, USA), 1 % L-glutamine (Gibco, USA), and 1 % penicillin-streptomycin (Gibco, USA). Culture media were changed every 1 days, and cells were passaged when confluents with 0.25 % Trypsin-EDTA (GenDEPOT, USA). First to four passage cells were used in experiments. CRL-4003 and human umbilical vein endothelial cell (HUVEC) were maintained as previously described 32.

### Transplantation of organoids and isolated mitochondria into the uterine cavity of AS mouse

Two days after AS modelling, female mice were anaesthetized and a small incision onto abdomen was made to gain access to uterine cavity. Prior to the transplantation of endometrial organoids, approximately 10 ul of matrigel was infused into the uterine horns for the temporal blocking and preventing the leakage of transplanted organoids. Subsequently, organoids (1x10^6^ cells) were slowly delivered into the uterine horn using a 31-gauge syringe. All mice were sacrificed at 7, 14 and 21 days after transplantation for further analyses. Mitochondria were isolated from mouse endometrial organoids (1x10^7^ cells) by using the mitochondria isolation kit (Cat. #89874, Thermo, USA). Successful isolation of mitochondria was confirmed by detecting mitochondrial DNA using gel electrophoresis. Isolated mitochondria were delivered to the uterine horn for further analyses in the same manner with organoid transplantation.

### Histology and immunohistochemistry

Tissues and organoids were fixed with 4 % paraformaldehyde, respectively embedded in paraffin. Paraffin-embedded sections (5 μm) were deparaffinized in Histoclear (National Diagnostics, USA), hydrated through a descending gradient of ethanol (100-70), and then stained with H&E and using a Masson's trichrome staining kit (Agilent, USA). For immunohistochemistry, antigen retrieval was performed in sodium citrate buffer (pH 6) for 30 min in the microwave. Samples were blocked and overnight at 4 °C with primary antibodies to α-SMA (Abcam, UK), MUC1 (Abcam, UK), STEM121 (Takara, Japan), h-MITO (Novus, USA), COL1A1 (Santacruz, USA) and VEGF (Invitrogen, USA) for overnight and further incubated with biotinylated goat anti-rabbit or mouse secondary antibodies (Vector laboratories, USA) for 1 h at room temperature. Localizations of each protein were identified by diaminobenzidine tetrachloride reaction (Vector laboratories, USA).

### Immunofluorescence and microscopy

Immunofluorescence staining was performed as previously described 33. Briefly, CD31 (Abcam, UK), Ki67 (BD Biosciences, USA), E-CADHERIN (Santacruz, USA), CK14 (Abcam, UK), ER (GeneTex, USA), TOMM20 (Abcam, UK) and GFP (Santacruz, USA) was detected in fixed and paraffin embedded-endometrial tissue or organoids sections. At last, nuclei were stained with DAPI (Sigma, USA). Images were observed by Mica microhub and Thunder imager (Leica Microsystems, Germany) and analyzed by LAS X software (Leica Microsystems, Germany).

### Quantitative RT-PCR-based analysis of mRNA expression

Whole uteri were homogenized in Labozol (Cosmo, South Korea) and the complementary DNAs were synthesized as previously described 33. RT-qPCR was performed on cDNA to assess the expression of genes of interest with SYBR Green (Enzynomics, South Korea). Experimental gene expression data were normalized to the housekeeping gene *Rpl7* and *Actb*. Primer sequence pairs used in the experiments are shown in Table S1.

### Flow cytometry

Culture organoids were harvested using cold PBS and dissociated into single cells by incubation with TrypLE Express. Single cell suspensions were washed with FACS buffer (0.5 % BSA in 1 X PBS) and stained with anti-CD326 (APC/human; Miltenyi, Germany), anti-CD326 (APC/mouse; Miltenyi, Germany), anti-CD44 (APC/mouse; Miltenyi, Germany) and anti-rat IgG2b (APC; Miltenyi, Germany), anti-mouse IgG1 (PE; Miltenyi, Germany), anti-human IgG1 (APC; Miltenyi, Germany), anti-human IgG1 (PE; Miltenyi, Germany) for 30 min. Cells were washed 3 times and finally resuspended in 400 μl of FACS buffer. All samples were run on a Cyto-FLEX-Analyzer (Beckman coulter, USA) and data were analyzed by CytExpert software (Beckman coulter, USA).

### Fertility assessment

AS-induced mice were mated with same strain male at a ratio of 1:1 for overnight. The vaginal plug was checked in the next morning and defined as pregnancy day 1. At pregnancy day 14, uteri were harvested and subsequently the total number of implantation sites, fetus and placenta weight were recorded. In the case of mice which were undergone transplantation, they were mated at day 21 after transplantation and further analyses were performed in the same way.

### Hydrogel and cell loading in endometrium-on-a-chip

The microfluidic devices were designed and fabricated as previously described with minor modifications 34. In brief, polydimethylsiloxane (PDMS; Dow corning, USA) was thoroughly mixed in 10:1 ratio to curing agent and poured into custom-made micro-patterned SU-8 (MicroChem, USA) molds. After curing process, PDMS was removed from molds using edged knife and cell injection ports and culture medium reservoirs were created by biopsy punch, respectively. Following plasma treatment, device surface obtained hydrophobic state and underwent sterilization before use. For cell loading into devices, HUVEC and mESCs (5 x 10^6^ cells/mL) were mixed with fibrin gel solution consists of 2.5 mg/mL fibrinogen (Sigma, USA) with 0.15 U/mL aprotinin (Sigma, USA) and then rapidly loaded in each channel. The device was incubated at 37 °C with 5 % CO_2_. To mimic the AS conditions, devices were treated with or without 5 ng/ml TGF-β (R&D Systems, USA) in EBM basal medium (Lonza, Swiss) for 24 h. The upper reservoirs of device were filled with EGM2 medium (Lonza, Swiss) and then aspirated gently from the lower reservoirs to wet the hydrophobic media channels. Next day, Mitotracker (Invitrogen, USA) stained mouse endometrial organoids were loaded onto stromal channel as an epithelium layer (6 x 10^6^ cells/mL). To make the evenly positioned layer of organoids on the gel surface, the device was rotated by 90 º for 30 min with the stroma channel facing down. The stromal channel was filled with organoid culture medium, and endothelial channel was filled with EGM2 medium supplemented with 1 nM sphingosine 1-phosphate (Sigma, USA), 100 ng/ml VEGF-A (R&D Systems, USA), 50 ng/ml VEGF-C (R&D Systems, USA) and 50 ng/ml bFGF (R&D Systems, USA). Finally, devices were fixed at 0, 1 and 24 h after organoids loading. Images were observed under confocal microscope Zeiss LSM880 (Carl Zeiss, Germany) and further analyses were performed by ZEN black software (Carl Zeiss, Germany).

### Transmigration assay

CRL-4003 (2 x 10^5^ cells/ml) were seeded in a 24-well plate and incubated at 37˚C for 24 h. Next, mouse endometrial organoids (1.5 x 10^6^ cells/ml) were placed in the upper compartment of transwell (Corning, USA) and co-cultured for the next 2 days. To identify migrated mitochondria from mouse endometrial organoids to CRL-4003, CRL-4003 were harvested with trypsin-EDTA and sampled for digital PCR analysis.

### Digital RT-PCR analysis

To detect the small copy number alterations, RT-dPCR analyses were conducted. Two microgram of cDNA, 400 nM of each primer, and 13.3 μl of 4x EG PCR master mix (Qiagen, Germany) were mixed to a final volume of 40 μl and prepared into Nanoplate 26k 24-well (Qiagen, Germany), which were sub-divided into 26,000 partitions. Experiments were performed by Qiacuity One, 5plex (Qiagen, Germany). The fluorescent signals from each sample were detected and calculated by auto-setting thresholds compared to non-template control (NTC/negative control).

### Mitochondrial membrane potential assessment

FCCP (Abcam, USA), Taxol (Selleckchem, USA) and Rotenone (Sigma, USA) were applied to induce mitochondria dysfunction in mouse endometrial organoids. Organoids (1x10^6^ cells/well) were treated with FCCP (25 uM, 24h), Taxol (10 nM, 24h), and Rotenone (100 nM, 48h). After treatments, organoids were dissociated into single cells, and then incubated with TMRE (Abcam, USA) at a final concentration of 250 nM for 30 min. Active mitochondria were detected by Cyto-FLEX-Analyzer for 488 nm at excitation and 575 nm at emission.

### Co-culture of endometrial organoid-originating mitochondria with mouse ESC

To investigate mitochondria mediated-metabolic recovery in the AS state, mESCs (5x10^4^ cells) were treated with or without 5 ng/ml of TGF-β for 24 h. Next, isolated mitochondria from mouse endometrial organoids were added onto mESCs and maintained for the next 48 h.

### ATP levels measurement

For ATP level measurements, a luminescent ATP detection assay kit (Abcam, USA) used according to manufacturer's instructions. Briefly, co-cultured samples were gently collected and centrifuged. Purified islets were lysed, and luciferin-luciferase solution was added. Following incubation, intracellular ATP concentration was measured by Spectramax ID5 (Molecular Devices, USA).

### Statistical analyses

Comparison groups were analyzed with unpaired t-test for parametric distributions. For multiple comparisons, the ordinary one-way ANOVA analysis with Dunnett's multiple comparison test or two-way ANOVA analysis with Tukey's multiple comparisons test were used. For all cases, a P value that was < 0.05 was considered statistically significant (P < 0.05 (*), P < 0.01 (**), P < 0.001 (***) and P < 0.0001 (****)).

## Results

### Murine model of AS recapitulates the pathophysiological features of human AS

**Figure 1:**
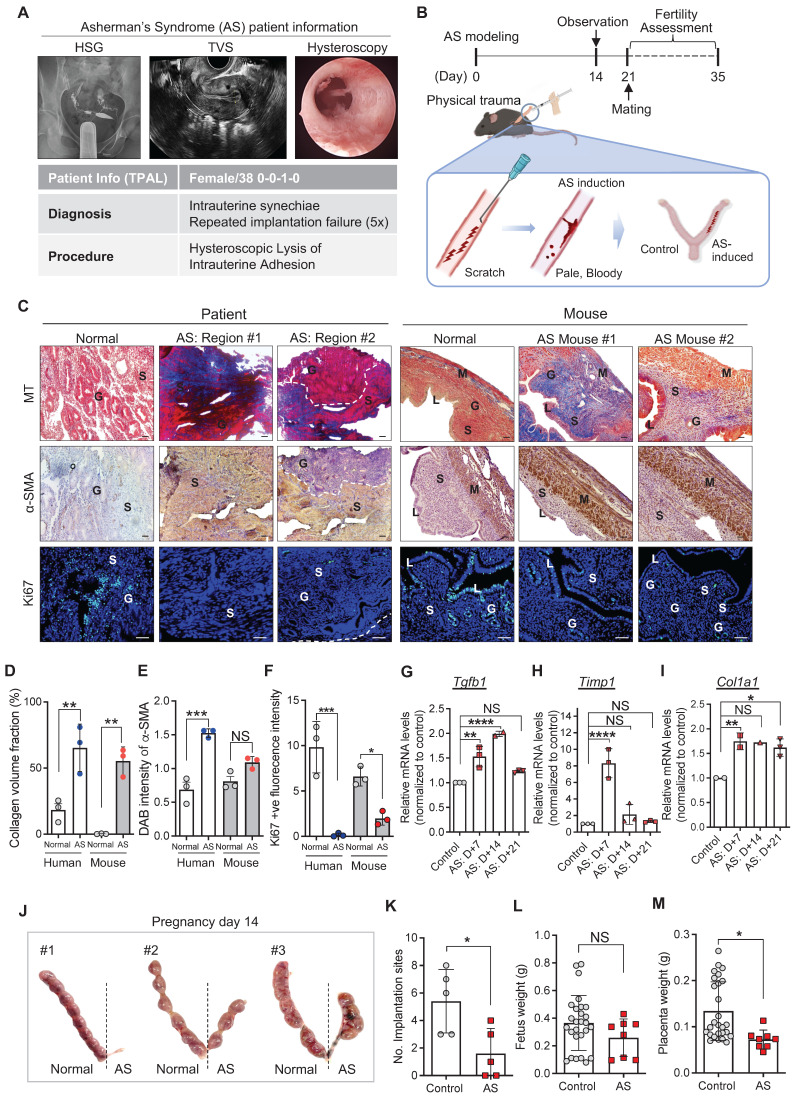
**Murine model of AS recapitulates the pathophysiological features of human AS. (A)** Hysterosalpingography (HSG), transvaginal ultrasound (TVS), and hysteroscopy images of patient with AS (n=1). T-P-A-L stands for term birth-preterm birth-abortion-living baby. **(B)** A schematic diagram of experimental design displaying the induction of AS in mice and its validation. **(C)** Images of Masson's trichrome (MT), α-SMA, and Ki67 (green) staining in endometrial tissues from normal (n=1) and patient with AS (n=1), and mouse endometrial tissues from normal (n=3) and AS-induced uteri (n=3). DAPI (blue) was used for nuclei staining. Scale bar; 25um. S: stroma, G: glandular epithelium, L: luminal epithelium, M: myometrium. Quantification of collagen volume fraction **(D)**, α-SMA intensity **(E)**, and Ki67^+^ fluorescence intensity **(F)** in all groups. QRT-PCR analyses of *Tgfb1* **(G)**, *Timp1* **(H)**, and *Col1a1*** (I)** mRNA expression in the AS-induced mouse uteri at indicated days (7, 14 and 21) compared to normal mouse uteri. (Total number of mice used=48; 3 mice per group; triplicates) **(J)** Images of uteri with implantation sites on pregnancy day 14 (n=5). The number of implantation sites **(K)**, fetus **(L)** and placenta weight **(M)** were counted in each horn and quantified to normal uterine horn in following graphs. Data were expressed as mean ± SD, analyzed using the ordinary two-way ANOVA with Turkey's multiple comparisons test **(D-F)** or ordinary one-way ANOVA with Dunnett's multiple comparisons test** (G-I)** or unpaired t-test** (K-M)** including P-values (*<0.05, **<0.01, ***<0.001, ****<0.0001, NS; not significant).

Endometrial tissues were obtained from patients diagnosed as normal or with AS and underwent endometrial biopsy for subfertility complaints. The patient with AS was a 38-year-old nulligravid with a medical history of abortion (once) and was diagnosed with RIF (five-time implantation failure). Hysterosalpingography (HSG) images showed no congenital uterine anomalies and tubal ligation, supporting the diagnosis made using transvaginal ultrasound (TVS). However, hysteroscopy images revealed severe intrauterine synechiae in the endometrial cavity (**Figure 1A**). To mimic these endometrial conditions of the patient with AS in mice, we established an experimentally-induced murine model of AS by inducing physical trauma through repeated mechanical curettage to the uterine horns (**Figure 1B**), adopted and modified from previous reports 25, 30, 31. For the validation of successful modeling, damaged uterine tissues, obtained 2, 7, 14, and 21 days after AS induction, were analyzed and compared with endometrial tissues from the patients with AS (**Figure 1B-C** and **Figure S1**). In order to control the issues from various estrous cycle, AS induction was conducted in one side of uterine cavities and the other side was used as a control (**Figure 1B**). Histological evaluation revealed that fibrotic lesions were appeared with severe collagen accumulation and activated fibroblasts displaying a highly expressed blue staining for Masson's trichrome (MT) and α-SMA (**Figure 1C-E and Figure S1A-C** Moreover, the number of Ki67-positive cells was dramatically decreased in the AS-induced mouse endometrium, which was consistently observed in human endometrial tissues with AS compared with normal tissues (**Figure 1C and 1F, and Figure S1A and S1D**). This is supported by the expression profiles of the molecular markers of fibrosis, including *Tgfb1*, *Timp1*, and *Col1a1* (**Figure 1G-I**). The overall analyses of physically traumatized uteri appeared to maintain for 3 weeks after the experimental induction of AS. Especially, a significantly lower number of embryo implantation sites recorded at pregnancy day 14 (p=0.0040; n of mice=5; n of implantation sites=35 (control: 27, AS:8)) was observed in the AS-induced endometrium than in the control mice (**Figure 1B and J-K**). Of note, even though the fetal weight was observed to be similar in the control and AS-induced endometrium (embryo resorptions were excluded), morphologically retarded fetuses, placenta, and more resorption sites were observed in mice with AS (**Figure 1L-M and Figure S1E**), which is a key feature observed in patients with AS 35.

### Endometrium-derived organoids are successfully engrafted in a mouse model of AS

**Figure 2:**
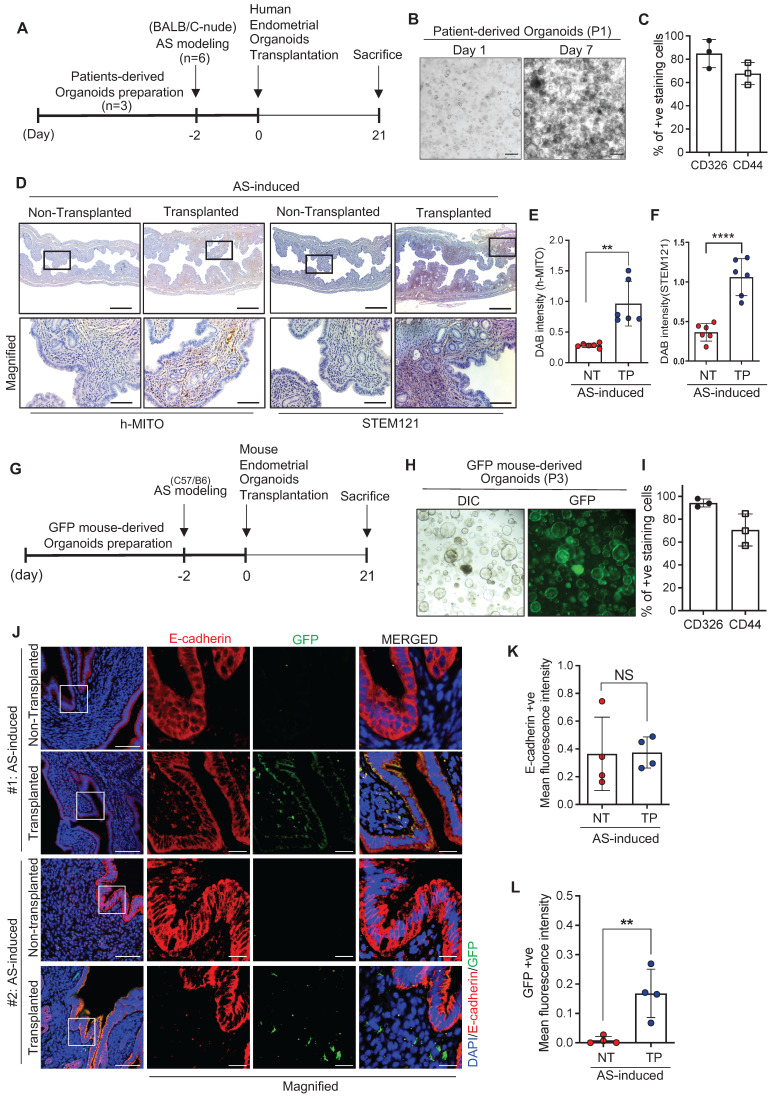
**Endometrium-derived organoids are successfully engrafted in a mouse model of AS. (A)** An experimental design of human endometrial organoid transplantation to the AS-induced uteri of BALB/C mice.** (B)** Brightfield images of cultured organoids used for transplantation. Scale bar; 25um. **(C)** Expression profiles of CD326 and CD44 in cultured organoids used for transplantation.** (D)** Immunohistochemical analyses of h-MITO and STEM121 in human endometrial organoids-transplanted AS-induced endometrium compared with non-transplanted AS-induced endometrium. Scale bar; 25um. Expressions of h-MITO or STEM121 intensity were quantified in graphs in **(E)** and** (F)**, Total number of mice used=4; 2 mice per group; duplicates).** (G)** An experimental design of GFP**^+^** mouse endometrial organoid transplantation to the AS-induced uteri of C57/B6 mice.** (H)** DIC and GFP fluorescent images of cultured organoids used for transplantation.** (I)** Expression profiles of CD326 and CD44 in cultured organoids used for transplantation. **(J)** Co-immunofluorescent analyses of E-CADHERIN (red) and GFP (green) with DAPI (blue) in GFP**^+^** mouse endometrial organoids-transplanted AS-induced endometrium compared with non-transplanted AS-induced endometrium. Scale bar; 50um, 10um (Magnified). E-cadherin +ve or GFP +ve intensity was quantified in graphs of **(K)** and **(L)**, Total number of mice used=6; Total number of organoid transplantations=3; Total number of human samples used=3; 2 mice per transplantation; triplicates). Data were expressed as mean ± SD, analyzed using the unpaired t test including P-values (*<0.05, **<0.01, ***<0.001, ****<0.0001, NS; not significant).

As a therapeutic strategy for endometrial regeneration, we generated human endometrial organoids using biopsied endometrial tissues obtained from four patients who were admitted to the CHA Fertility Center Bundang and diagnosed with normal endometrial conditions. Obtained tissues were mechanically and enzymatically dissociated and filtered to include the epithelial cell-enriched fraction. Afterwards, isolates collected were embedded with collagen droplets to self-organize into organoid-like structures in an endometrial organoid culture medium (for the full composition was adopted from previous reports) 27, 29 (**Figure S2A-G**). Even though there was a variation among donors, the established organoids were generally well-expanded and passaged up to six times with a 3.10-fold increase in the number of cells (**Figure S2A-B**). The cultured endometrial organoids expressed CD326 and CD44, well-known markers of luminal and glandular epithelium (CD326; EpCAM) and stemness (CD44), which displayed a robust increase in the number of positively stained cells (CD326, 5.00-fold; CD44, 2.25-fold) as the passage number increased (**Figure S2C**). Moreover, the established organoids exhibited a similarity with parental tissues. MUC1 and E-cadherin, markers for glandular epithelium, were strongly expressed in parental tissues and organoids, and localized at the basal membrane, reflecting an intact epithelial polarity—a key feature observed *in vivo*. Furthermore, estrogen receptor α (ERα) was maintained in cultured organoids similar to the tissues. However, Ki67 was more abundant in the cultured organoids than in the tissues, implicating more proliferative cells were only remained to form endometrial organoids during the culture period after dissociation from the parental tissues (**Figure S2D**). This led us to examine the characteristics of established endometrial organoids, the mRNA expression levels of surrogate molecular markers of epithelial and stromal cells (*CDH1*, *EPCAM*, and *VIM*) were measured. Significantly higher expression of *CDH1* (3.3-fold) and *EPCAM* (1.9-fold) and lower expression of *VIM* (0.16-fold) were observed in the cultured organoids compared to their parental tissues, revealing that the epithelial fraction dissociated from the parental endometrial tissue becomes proportionally enriched whereas stromal fraction becomes disappeared during the organoid culture period (**Figure S2E-G**). To determine the engraftment potential of the organoids onto the damaged endometrium, we transplanted human endometrium-derived organoids (1x10^6^ cells) into one side of cavities of the AS-induced endometrium of BALB/c-nude mice 36, 37 and non-transplanted the other side was used as a control (**Figure 2A**). The cells used for transplantation were well-expanded and displayed 84.84% (±11.16) of CD326-positive staining and 67.6% (±9.40) of CD44-positive staining (**Figure 2B-C**). For more efficient transplantation, organoids were transplanted within matrigel (1:1 ratio) 2 days after AS induction (**Figure 2A**). Successful engraftment of human endometrial organoids onto a recipient mouse endometrium was clearly detected using antibodies for human mitochondria (h-MITO) and the cytosol (STEM121) on day 21 after transplantation (**Figure 2D-F**).

Furthermore, mouse endometrial organoids were generated from the uterine tissues of C57BL/6-Tg (CAG-EGFP) 1Osb/J mice, which displayed a 6.7-fold increase in cell expandability up to passage 15, with features of most of the cells expressing CD326 (99%) and CD44 (97%), indicating that epithelial progenitor cells became enriched during the culture periods (**Figure S2H-J**). Mouse endometrial organoids exhibited a similarity with parental tissues like observed in human organoids (**Figure S2K**). Successful transplantation of mouse endometrial organoids, displaying 94.3% (±3.7) of CD326- and 70.6% (±14.4) of CD44-positve features (**Figure 2G-I**), to the uterine cavity of C57/BL6 mice was validated using the immunofluorescence analysis of enhanced green fluorescent protein (EGFP) displaying a high level of GFP-positive signals in mouse endometrial organoids-transplanted endometrium of a recipient mouse compared with non-transplanted endometrium (**Figure 2J-L**).

Moreover, transplanted endometrial organoid-derived GFP-positive signals were detected 3 months after transplantation, displaying a reduced pattern even though there was no statistical significance when compared to GFP signals observed at 2-week time point after transplantation (**Figure S3A-C**). This might implicate that the effect mediated by one time transplantation of endometrial organoids might not be transient or temporally constrained.

### Engrafted endometrial organoids induce endometrial regeneration in mice with AS

**Figure 3:**
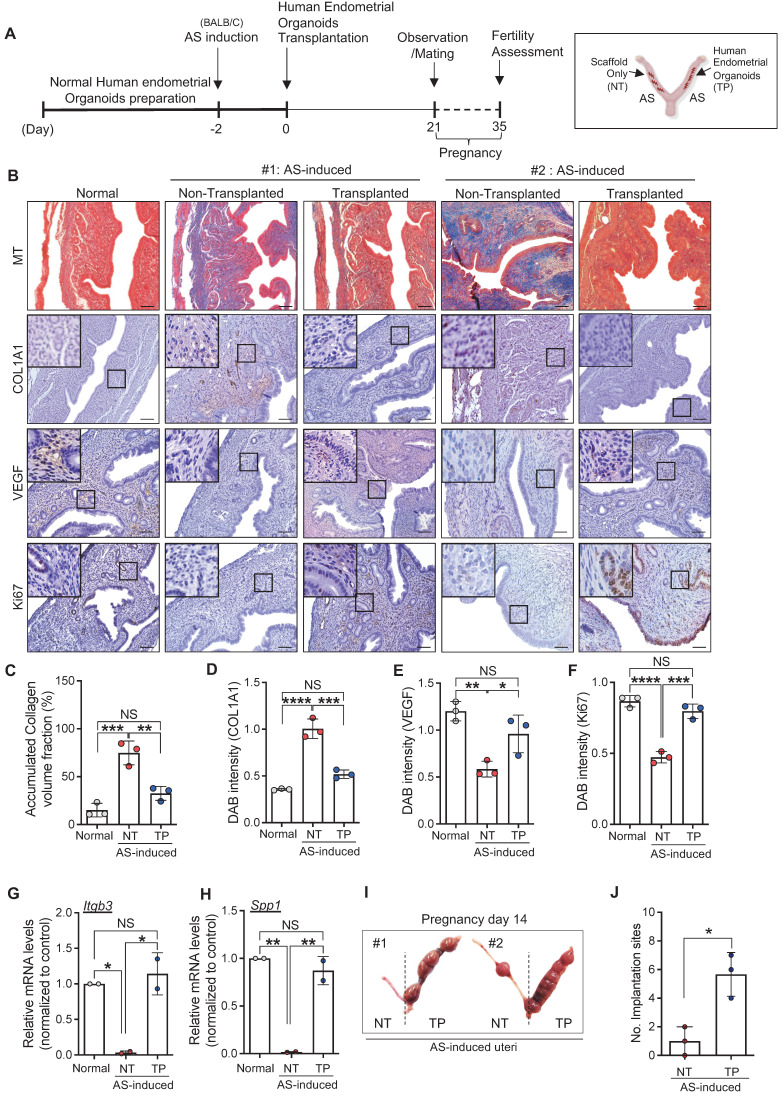
**Engrafted endometrial organoids induce endometrial regeneration in mice with AS. (A)** An experimental design of human endometrial organoid transplantation to AS-induced uteri of BALB/C mice and further analyses including fertility assessments.** (B)** Immunohistochemical analyses of MT, COL1A1, VEGF, and Ki67 in normal endometrium (Normal), AS-induced endometrium (non-transplanted) and organoid-transplanted AS-induced endometrium (Transplanted). Each indicated region was magnified. Scale bar; 25um. Expression levels of collagen volume **(C)**, intensity of COL1A1 **(D)**, VEGF **(E)**, and Ki67 **(F)** were quantified (Total number of mice=6; 2 mice per group; triplicates). QRT-PCR analyses of endometrial receptivity related markers (*Itgb3* **(G)** and* Spp1* **(H)**) in normal endometrium (Normal), AS-induced endometrium (non-transplanted) and organoid-transplanted AS-induced endometrium (Transplanted) (Total number of mice=4; 2 mice per group; duplicates).** (I)** Representative images of uteri with implantation sites on pregnancy day 14. The number of implantation sites was quantified in a graph **(J)**. Data were expressed as mean ± SD, analyzed using the ordinary one-way ANOVA with Dunnett's multiple comparisons test **(C-H)** or unpaired t-test **(J)** including P-values (*<0.05, **<0.01, ***<0.001, ****<0.0001, NS; not significant).

We next investigated the therapeutic effects of engrafted endometrial organoids in mice with AS. The uteri were harvested at 21 days after transplantation (**Figure 3A**). Immunohistochemical analyses revealed that the structurally damaged endometrium with severely accumulated collagen was significantly recovered by the engraftment of human endometrial organoids (accumulated collagen volume: 0.49-fold, COL1A1: 0.52-fold) (**Figure 3B-D**). Strongly reduced expressions of vascular endothelial growth factor (VEGF) (1.64-fold) and Ki67 (1.68-fold) in the AS-induced endometrium were restored back to the levels observed in normal endometrium by endometrial organoid engraftment (**Figure 3B, 3E-F**). Intriguingly, the mRNA levels of endometrial receptivity-related markers (*Itgb3, Spp1*) showed dramatic elevations after human endometrial organoid transplantation (*Itgb3*, 34.15-fold; *Spp1*, 53.02-fold), which was similar to the levels detected in the normal endometrium (**Figure 3G-H**). This is fully supported by significantly increased number of implantation sites in human endometrial organoid-engrafted AS-induced uteri compared with non-transplanted AS-induced uteri, suggesting that engrafted endometrial organoids alleviated the AS-induced damaged endometrial environment to more favorable to embryo implantation and pregnancy (**Figure 3I-J**). Transplantation of mouse endometrial organoids to the endometrium of AS mice showed effects similar to those observed in human endometrial organoid-transplanted AS endometrium, including dramatic recovery of fibrotic lesions with significantly decreased collagen accumulation (**Figure S4A-B**). Notably, the endometrial organoid-transplanted endometrium exhibited re-vascularization and re-proliferation with an increased expression of CD31 and Ki67 expression within the endometrial functionalis (**Figure S4C-E**). In addition, the total number of implantation sites was significantly higher in organoids-transplanted AS-induced uteri than in the non-transplanted AS-induced uteri (p=0.0075) (**Figure S4F-G**), which was consistently shown in the comparisons of litter sizes (**Figure S4H-I**). Endometrial organoid transplantation fully recovered reproduction capability of mice with AS endometrium in which significantly reduced the numbers of implantation sites and litters were observed to the levels shown in health control (Normal vs. AS-NT; p=0.0194, AS-NT vs. AS-TP; p=0.0255). However, the weights of litters from the mothers with endometrial organoids-transplanted AS-induced endometrium remained similar to those from mothers with non-transplanted AS endometrium, which were significantly lower than weights of health litters (Normal vs. AS-NT; p=0.00004, Normal vs. AS-TP; p=0.00012, AS-NT vs. AS-TP; 0.0615) (**Figure S4J**).

### Engrafted endometrial organoids mediate alterations in the mitochondrial function of recipient endometrium

**Figure 4:**
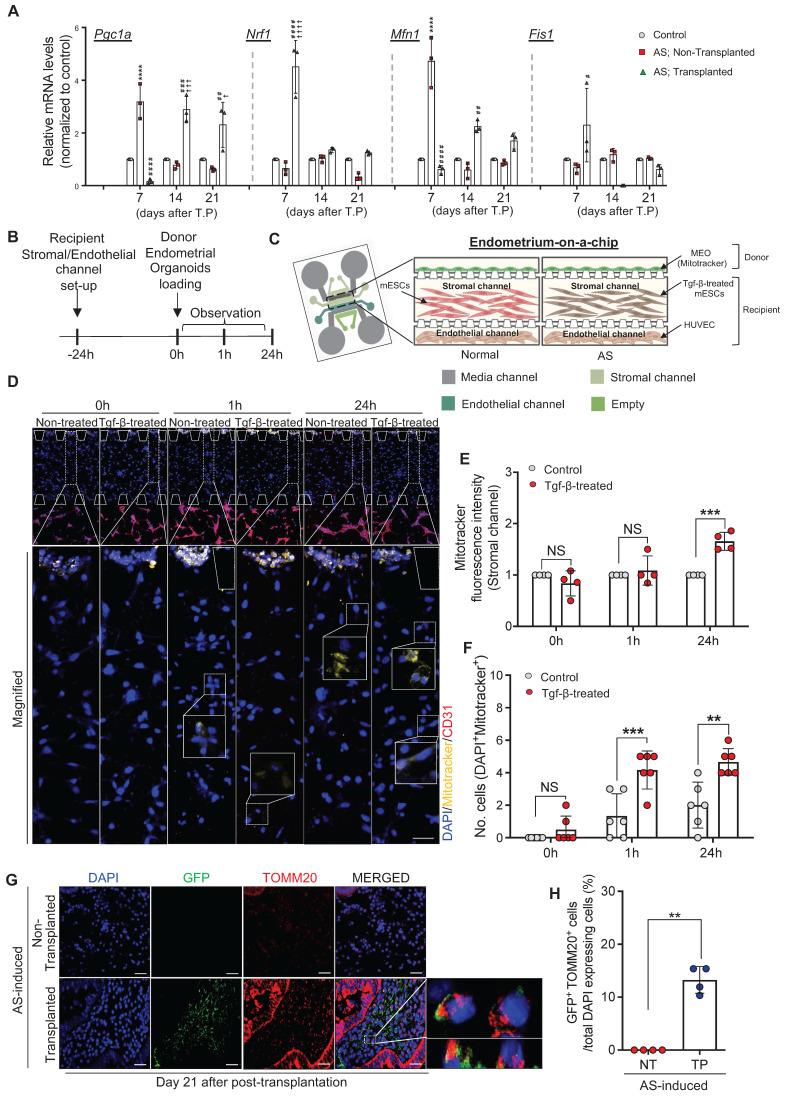
**Engrafted endometrial organoids mediate alterations in the mitochondrial function of recipient endometrium. (A)** QRT-PCR analyses of mitochondrial function related markers (*Pgc1a*,* Nrf1*,* Mfn1*, and *Fis1*) in normal endometrium (Normal), AS-induced endometrium (non-transplanted) and organoid-transplanted AS-induced endometrium (Transplanted) at indicated days (Day 7, 14, and 21) (Total number of mice=54; 3 mice per group (normal/non-transplanted, transplanted) for each day; triplicates). Data were expressed as mean ± SD, analyzed using the ordinary two-way ANOVA with Turkey's multiple comparisons test. (****; p<0.001 normal endometrium vs. non-transplanted endometrium, *####*;* p<0.001, ###*;* p<0.005, ##*;* p<0.01 and #; p<0.05* non-transplanted endometrium vs. organoids-transplanted endometrium, *††††*;* p<0.001, †††*;* p<0.005, †*;* p<0.05* normal endometrium vs. organoids-transplanted endometrium). **(B)** An experimental design of cell placement in an endometrium-on a-chip. **(C)** A schematic diagram of microfluidics design mimicking normal and AS-induced endometrium. **(D)** Immunofluorescent analyses of CD31 (red), Mitotracker (yellow) and DAPI (blue) in each layer of microfluidics device (triplicates). Magnified images indicated by dot lined-white boxes were shown in lower panels. Straight lined-white boxes in lower panels indicate transferred mitochondria. Scale bar; upper: 200um, lower: 50um.** (E)** Mitotracker fluorescence intensity in stromal channels of each group was quantified.** (F)** Number of DAPI^+^ and Mitotracker^+^ cells observed in stromal channels of each group was quantified.** (G)** Co-immunofluorescent analyses of GFP (green) and TOMM20 (red) with DAPI (blue) in organoid-transplanted AS-induced endometrium (TP) compared with non-transplanted AS-induced endometrium (NT). Scale bar; 50um. The ratio of co-localization of GFP and TOMM20 normalized to DAPI were displayed in **(H)** (n=4). Data were expressed as mean ± SD, analyzed using the ordinary two-way ANOVA with Turkey's multiple comparisons test **(A, E-F)** or unpaired t test** (H)** including P-values (*<0.05, **<0.01, ***<0.001, ****<0.0001, NS; not significant).

Dysfunctional mitochondria disrupt normal cellular biological processes by increasing ROS generation and altering bioenergetics, which are tightly associated with various fibrotic diseases often accompanied by chronic inflammation 38-40. This led us to seek whether the dramatic recovery of the AS-induced damaged endometrium with endometrial organoid transplantation was instigated by alterations in the mitochondrial function of the recipient endometrium. We first examined changes in the expression of mitochondrial function-related genes including *Pgc1a, Nrf1*,* Mfn1*, and *Fis1* in GFP mouse-derived endometrial organoids-transplanted AS-induced recipient endometrium on days 7, 14, and 21 compared with non-treated control or non-transplanted AS-induced endometrium (**Figure 4A**). Pgc1-α, a master regulator of mitochondrial biogenesis, decreases in the fibrogenic state 41, 42. However, a dramatically elevated level of *Pgc1a* was detected in the AS-induced endometrium on day 7 compared to normal endometrium (3.17-fold) and afterwards it was decreased back to the level observed in normal endometrium.

This might imply a selective mechanism to be prioritized for the acute injury induced by physical trauma rather than dynamic changes in mitochondrial biogenesis against fibrotic progression during the early period (day 7), and as the inflammatory response continued, *Pgc1a* appeared to be down-regulated to response to the chronic state. However, endometrial organoid transplantation drove a rapid response to fibrotic progression by reducing recipient* Pgc1a* expression compared to that observed in the early period (0.15-fold). On days 14 and 21 after transplantation, *Pgc1a* was highly expressed (day 14, 2.88-fold; day 21, 2.60-fold), implying that the cellular activities of the AS endometrium were restored back to normal status by the engrafted endometrial organoids. Moreover, Nrf1, a key transcription factor that regulates mitochondrial function, was abruptly increased in the endometrial organoid transplanted-AS endometrium on day 7 after transplantation. On days 14 and 21, little difference in *Nrf1* expression were observed in all groups. In mammals, dysfunctional mitochondria are removed by mitophagy through sophisticated network regulated by the dynamic balance between fission and fusion, thereby controlling the number of mitochondria 43. Likewise, Mfn1, a representative marker of mitochondrial fusion, showed a similar pattern of expression in all conditions, and Fis1, a representative marker of mitochondrial fission, exhibited exactly reversed expression pattern. In the AS endometrium on day 7, we observed exceptionally increased mRNA levels of *Mfn1* (4.72-fold) and decreased *Fis1* (0.67-fold) compared with the normal endometrium and after endometrial organoid transplantation expressions of both *Mfn1* and *Fis1* were reversely (*Mfn1*, 0.61-fold; *Fis1*, 2.29-fold) (**Figure 4A**). Collectively, these results suggest that AS induction with repeated mechanical traumatization upsets mitochondrial dynamics in the recipient endometrium and subsequent endometrial organoid transplantation reformats destroyed dynamics back to the normal state balancing mitochondrial fission and fusion leading to removal of dysfunctional mitochondria.

We next asked whether these mitochondrial alterations occurred in the recipient endometrium were induced by mitochondria originating from the transferred endometrial organoids. To recapitulate the stratified structure of the endometrium we developed a three-dimensional microfluidic system that mimics the vascularized endometrial stromal layer containing the stromal (mouse endometrial stromal cells, mESC) and endothelial channels (human umbilical vein endothelial cells). Cells treated with TGF-β for 24 h to mimic AS-induced fibrosis displayed increased COL1A1 expression (**Figure S5A**). Mitotracker stained-organoids were loaded on top of the stromal layer channel to replicate the endometrial organoid transplantation *in vivo* (**Figure 4B-C and Figure S5B**). Mitotracker fluorescence intensity was measured to detect organoid-originating mitochondrial movement by assessing the whole region of stromal channel using Image J. Movements of endometrial organoid-originating mitochondria were observed in the stromal layer 1 h after loading the endometrial organoids and more active movement was detected in TGF-β-treated mESC layer compared with non-treated cells at 24 h, displaying 2.18-fold higher Mitotracker fluorescence intensity in the stromal channel (**Figure 4D-E**). Of note, a higher number of Mitotracker-stained mitochondria was detected within TGF-β-treated stromal cells indicating that transferred mitochondria prefer to move into damaged cells than normal cells (**Figure 4F**). To support these findings, we additionally conducted a trans-well migration assay in which mouse endometrial organoids and human endometrial stromal cells (CRL-4003) were plated in the top and bottom chambers, respectively (**Figure S5C-D**). After 48 h of co-culture, the levels of mouse nuclear DNA (mNuDNA) and mitochondrial DNA (mMtDNA) in the stromal layer were measured using digital polymerase chain reaction (PCR). These analyses revealed that the mitochondria from co-cultured endometrial organoids were transferred and migrated towards the stromal layer in bottom chamber exhibiting significantly increased levels of mMtDNA (1.27x10^4^-fold) (**Figure S5E-F**). Notably, there was no difference in the level of mNuDNA between mono-cultured and mouse endometrial organoids co-cultured stromal cells (**Figure S5G-H**), indicating mitochondrial transfer, not organoids themselves. Consistently, it has been observed that transferred mitochondria originating from GFP mouse-derived endometrial organoids were successfully implanted in the recipient endometrium with a strong co-localized signals of GFP and TOMM20 on 21 days after organoid transplantation (**Figure 4G-H**).

### Engrafted endometrial organoid-originated mitochondria are essential for endometrial organoids-mediated regenerative effects

**Figure 5:**
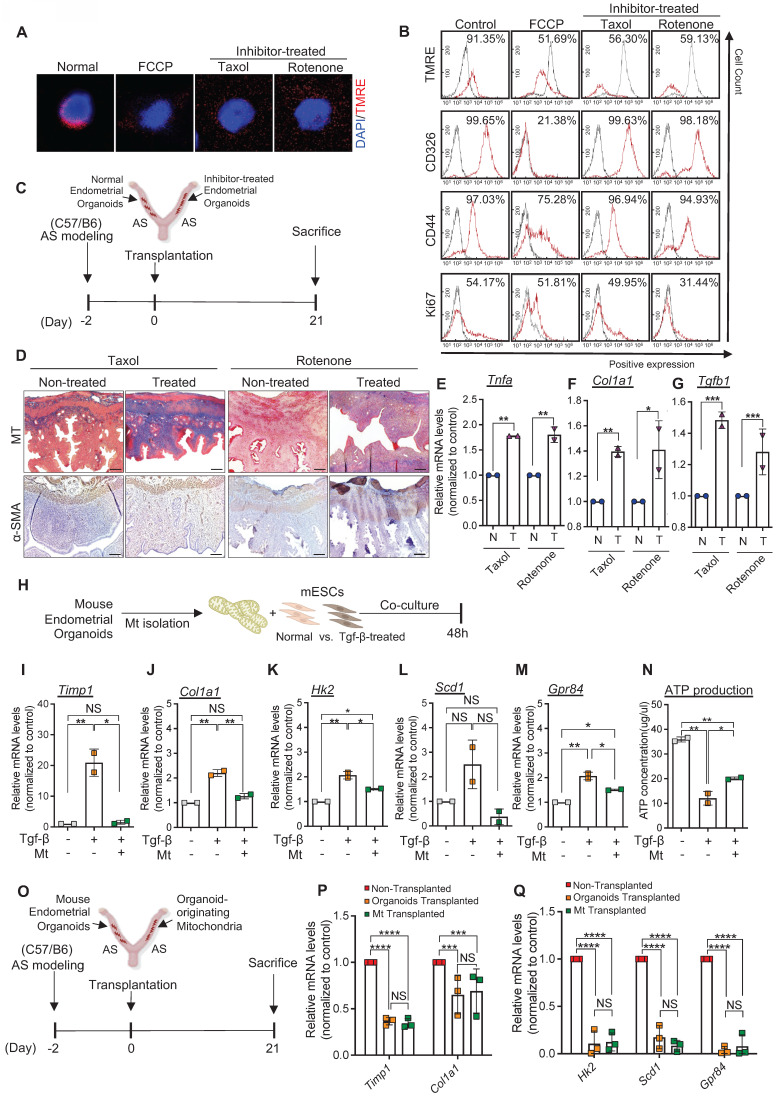
** Engrafted endometrial organoid-originated mitochondria are essential for endometrial organoids-mediated regenerative effects. (A)** Immunofluorescent analyses of TMRE (red) in mouse endometrial organoids treated with FCCP, Taxol, and Rotenone compared with non-treated normal group. Nuclei stained with DAPI (blue). Scale bar; 20um. **(B)** Flow cytometry analyses of TMRE, CD326, CD44, and Ki67 in FCCP, Taxol, or Rotenone-treated mouse endometrial organoids compared with control group. **(C)** An experimental design of transplantation of normal or inhibitor-treated mouse endometrial organoids to AS-induced uteri of C57/B6 mice. **(D)** Immunohistochemical analyses of MT and α-SMA of Taxol or Rotenone-treated endometrial organoid transplanted AS-induced endometrium compared with non-treated AS-induced group (n=3 per group). Scale bar; 25um. QRT-PCR analyses of *Tnfa* **(E)***, Col1a1* **(F)**, and *Tgfb* **(G)** in Taxol or Rotenone-treated endometrial organoid transplanted AS-induced endometrium compared with non-treated AS-induced group. **(H)** An experimental design of co-culture of non-treated or Tgf-β-treated mESCs with mitochondria isolated from normal mouse endometrial organoids. QRT-PCR analyses of *Timp1* **(I)***, Col1a1*** (J)**, *Hk2* **(K)**, *Scd1* **(L)**, and *Gpr84* **(M)** in each group. **(N)** Comparisons of ATP production in Tgf-β-treated mESCs co-cultured with mitochondria isolated from normal mouse endometrial organoids compared with mono-cultured Tgf-β-treated mESCs or non-treated mESCs. **(O)** An experimental design of transplantation of normal mouse endometrial organoids or mitochondria isolated from normal mouse endometrial organoids in AS-induced uteri of C57/B6 mice. **(P-Q)** QRT-PCR analyses of fibrosis related markers (*Timp1* and *Col1a1*), metabolite related markers (*Hk2, Scd1* and* Gpr84*) in mitochondria-transplanted or endometrial organoid-transplanted AS-induced endometrium compared with non-transplanted AS-induced endometrium (Total number of mice=6; 2 mice per group (non-transplanted, organoids-transplanted/organoids-transplanted, Mt-transplanted)). Data were expressed as mean ± SD, analyzed using the ordinary two-way ANOVA with Turkey's multiple comparisons test **(E-G**, **P-Q)** or one-way ANOVA with Dunnett's multiple comparisons test **(I-N)** including P-values (*<0.05, **<0.01, ***<0.001, ****<0.0001, NS; not significant).

We further explored whether mitochondria transferred from engrafted endometrial organoids to the AS-induced damaged endometrium mediated anti-fibrotic effects in the endometrium of a recipient mouse. To address this, Taxol and Rotenone, common inhibitors for functional mitochondria were used to artificially abrogate the function of mitochondria in endometrial organoids. Following the inhibitor treatments, abrogation of organoid-originating mitochondrial function was validated by measuring the penetration status of Tetramethyl rhodamine ethyl ester (TMRE) using immunofluorescence and flow cytometry. These analyses revealed that decreased TMRE expression in inhibitor-treated endometrial organoids, which showed similar TMRE levels observed in FCCP (positive control; carbonyl cyanide 4-(trifluoromethoxy)phenylhydrazone))-treated organoids (**Figure 5A**). However, the epithelial-enriched originality (CD326), stemness (CD44), and proliferative potentials were stably maintained in endometrial organoids even after Taxol or Rotenone treatments, implying that only mitochondrial function, rather than other critical features of the organoids, was disturbed by the inhibitors (**Figure 5B**). We next transplanted endometrial organoids with dysfunctional mitochondria into the cavity of the AS-induced endometrium and compared their therapeutic effects with those of transplanted organoids with functional mitochondria (**Figure 5C**). Immunohistochemical analyses of MT and α-SMA revealed that the Taxol or Rotenone-treated endometrial organoid-transplanted endometrium exhibited unrepaired endometrial conditions with dense collagen depositions all over the endometrium with significantly higher levels of *Tnfa*, *Col1a1* and *Tgfb1* compared with the control (no inhibitor treatment) endometrial organoids-transplanted endometrium (**Figure 5D-G**), which is similar to the features observed in non-transplanted AS endometrium (**Figure 3B-C**). These might suggest that endometrial organoids with dysfunctional mitochondria may attenuate the regenerative therapeutic effects of organoid transplantation. Therefore, we further investigated the specific roles of transferred mitochondria originating from engrafted endometrial organoids in the recovery of fibrotic lesions in the AS-induced endometrium. Mitochondria isolated from endometrial organoids were co-cultured with Tgf-β-pre-treated mESCs for 48 h *in vitro*. Expectedly, the fibrotic response was reduced displaying significant decreases of *Timp1* and *Col1a1* in mitochondria co-cultured cells compared with the Tgf-β-pre-treated mESC groups (**Figure 5H-J**). In addition, co-culture with mitochondria isolated from endometrial organoids stabilized Tgf-β-induced abrupt increase in *Hk2*, a crucial factor of glycolysis (**Figure 5K**). Moreover, Tgf-β-induced aberrant elevation in the expression levels of *Scd1* and *Gpr84*, markers for lipid metabolism, were restored back to the levels observed in non-treated mESCs with co-culture with mitochondria even though the changes in *Scd1* were not found to be significant and ATP production levels were partially recovered (**Figure 5L-N**). These findings were further validated *in vivo* (**Figure 5O-Q**). Mitochondria isolated from mouse endometrial organoids were administered into the AS-induced damaged uterine cavity and parental endometrial organoids were administered into the other side of the uterine cavity as a control (**Figure 5O**). Comparison between organoid-originating mitochondria- and parental organoid-transplanted endometria revealed that mRNA levels of *Timp1* and *Col1a1* showed similar levels in both groups, which are significantly decreased when compared with the non-treated AS-induced endometrium (**Figure 5P**). Moreover, the shift in mitochondrial metabolism was surprisingly balanced by administrating organoid-originating mitochondria or parental organoids demonstrating significant decrease in the markers of glycolysis (*Hk2*) and lipid metabolism (*Scd1* and *Gpr84*) (**Figure 5Q**).

## Discussion

Conventional treatments for AS aim to restore uterine fertility and parturition through surgical resection of adhesions and hormonal therapy 44. However, the severe trauma that frequently occurs during surgery affects the basal layer of the endometrium, leading to resistance to hormone treatments and regeneration failure, resulting in high recurrence rates of intrauterine adhesions 45. Various therapeutic strategies, particularly cell therapy, have been suggested to overcome these limitations 46-48. Therapies using autologous bone marrow-derived stem cells or hematopoietic stem cell injections have been suggested to improve and restore the endometrial function of patients with AS by decreasing intrauterine adhesion and increasing the endometrial thickness and angiogenesis 47, 49. However, despite extensive research on cell therapies, it is still unclear whether evidence-based effective and reproducible treatments that are possibly able to use in clinics are available to improve the structural and functional morphology of the AS endometrium.

In the present study, we have demonstrated that endometrial organoids derived from human or mouse endometrial tissues, which recapitulate the morphological and molecular features of the parental tissues, were successfully engrafted and dramatically repaired AS-induced damaged endometrium, significantly reducing fibrotic lesions and increasing fertility outcomes in mice. Three-dimensional assembloids of organ-specific cells, known as organoids, demonstrate a high degree of genetic stability, expandability, differentiation capacity, and self-renewal 22, 27, 28, consistent with our established endometrial tissue-derived organoids (**Figure 2 and S2**).

Several studies have reported that endometrial organoids might provide a novel tool for researching the dynamic epithelial biology underlying infertility and proliferative endometrial diseases including endometriosis and endometrial cancer 50. Furthermore, endometrial organoids containing endometrial epithelial progenitor cells expressing the stemness markers CD44 and LGR5 functionally respond to sex hormones and stimuli for decidualization 27. More beneficial effects of endometrial organoid transplantation compared to uses of stem cells including long-term survival rates and lower carcinogenic potentials have been suggested 51. Thus, as an alternative to traditionally cultured stem cells, organoids were intrigued huge interests in the field of regenerative medicine including female reproduction 52, 53. To understand the cellular and molecular mechanisms underlying the endometrial organoids-induced anti-fibrotic effects and regeneration of the AS-induced damaged endometrium, we artificially established a murine model of human AS by repeated mechanical traumatizing to induce key features of human AS, which was adopted and modified from previous reports 25, 30, 31. Consistently, our AS-induced endometrium demonstrated dramatically increased collagen accumulation, reduced endometrial proliferation, and unsynchronized receptivity with poor embryo implantation rates, recapitulating similar conditions of AS in patients (**Figure 1**).

In further, analyses of the intrauterine transplantation efficacy of human endometrial organoids revealed that the instilled organoids were successfully engrafted onto the endometrial layer of the immunodeficient recipient mouse, which was confirmed by detecting the localization of human-specific mitochondria and cytosol (**Figure 2**). Endometrial organoid transplantation increased the proliferative capacity and angiogenesis marker, VEGF expression in the recipient endometrium finally resulting in dramatically improved pregnancy outcomes (**Figure 3**), consistent with a previous report showing increased reproduction capability with promoted vascularization via mouse endometrial organoid transplantation in AS-induced damaged endometrial tissues in mice 51. However, together with the utilization of mouse-originating organoids, the precise cellular and molecular mechanisms underlying endometrial organoid-mediated tissue repair remain unclear.

In the present study, we have suggested that mitochondrial transfer from transplanted endometrial organoids to damaged endometrial cells is a key cellular mediator of antifibrotic endometrial regeneration (**Figure 4 and 5**). During the process of fibrotic lesion formation, it has been known that increase in mitochondrial fission and decrease in mitochondrial fusion are reciprocally occurred in response to excessive oxidative stress, leading to mitochondrial dysfunction in fibrotic cells 54, 55. This has been observed in the endometrium harvested 7 days after AS induction (**Figure 4A**). During this time there might have been acute responses to severe damage in the endometrium, resulting in more fluctuated behaviors of mitochondria 56-58. This, in turn, exacerbates the fibrotic process by promoting fibroblast activation, which is responsible for the overproduction of ECM proteins 18. Intriguingly, both non-treated and AS-induced endometrium displayed low levels of mitochondrial biogenesis and dynamics occurred during 14-21 days after AS-induction. In non-treated endometrium, little dynamics in mitochondria was detected because homeostasis might have been well-maintained already in a steady state, thus there might be no need for extra active mitochondrial biogenesis and dynamics 59. In a steady state, it is known that the level of mitochondrial fission and fusion is constantly well-balanced to reach an appropriate equilibrium and mitochondrial biogenesis is actively maintained at a low level 60. Whereas the loss of mitochondrial biogenic function, fusion, and fission in damaged mitochondria at chronic state of inflammation occurred during 14-21 days after AS-induction might lead the low level of mitochondrial function-related gene expressions 61-63. Furthermore, more dynamics in mitochondria observed in endometrial organoid-transplanted endometrium compared to other groups on day 14 and 21 might be due to more biogenic activities and fusion followed by mitochondrial repair and recovery induced by additionally transplanted organoid-originating functional mitochondria. We have demonstrated that mitochondria originating from transplanted endometrial organoids were physically transferred and integrated into damaged fibrotic cells and subsequently restored the mitochondrial function in the recipient (**Figure 4 and S5**). This implies that mitochondrial fusion occurred by integration of donor mitochondria with those of the recipient may protect fibrosis-induced damaged cells and restore the cellular function by sharing their contents including proteins and genetic materials. Indeed, mitochondria derived from stem cells have been reported to show a significant effect on tissue recovery via transfer towards damaged cells in various tissues, including the retina, lung, and bone marrow 40, 64, 65. Our three-dimensional microengineered vascularized endometrium confirmed spontaneous mitochondrial transfer from endometrial organoids to the recipient. Moreover, the mitochondrial transfer was occurred more effectively when the endometrial stroma was in a damaged state, which was fully supported by *in vivo* observations exhibiting the co-localization of TOM20 and EGFP in endometrial organoids transplanted AS-induced endometrium (**Figure 4**). Analyses of digitized quantification using digital PCR consistently demonstrated that only mitochondria rather than the organoids themselves migrated from the co-cultured endometrial organoids towards the stromal layer (**Figure S5**). It has been extensively studied that mitochondria migrate via damage-associated molecular patterns, including tunneling nanotubes (TNTs), extracellular vesicles (EVs), gap junction channels (GJCs), and full fusion 66-69. Previously, it has been reported that human mesenchymal stem cells co-cultured with mitochondria-depleted cells developed cytoplasmic projections towards the target cells for the movements of healthy mitochondria. Although it was not able to establish whether this mitochondrial movement was mediated by TNTs or uptake of EVs that budded from the donor cells, this reveals that mitochondrial movement involves an active cellular process rather than passive uptake of cellular fragments or organelles 70. Even though detailed mechanisms of mitochondrial movement have not been fully elucidated, in our study it has been clearly shown that organoid-originating mitochondria moved to the recipient cells in response to the molecular cues mediated by inflammatory factors or cytokines secreted from AS (or TGF-β treatment)-induced damaged environment, in which mitochondria were dysfunctional (**Figure 4D-H**). Furthermore, we showed that the transplantation of endometrial organoids with dysfunctional mitochondria failed to induce anti-fibrotic and anti-inflammatory effects, suggesting that functional mitochondria are essential for mediating anti-fibrosis and endometrial tissue repair via endometrial organoids transplantation for AS treatment.

Fibrosis is widely associated with altered cellular energy metabolism, including glucose and lipid metabolism, mitochondrial dysfunction, and excessive oxidative stress 71, 72. An aberrant increase in glycolysis and a decrease in oxidative phosphorylation cause a metabolic shift towards more glycolytic environment in affected cells. These alterations contribute to activation and proliferation of fibroblasts, responsible for the excessive production of ECM components that directly lead to fibrosis progression 73. Furthermore, it has been recently reported that integrating donor mitochondria into recipient cells incorporates an endogenous network of energy metabolism, rescuing the aerobic cellular respiration and ATP production 70. Our findings confirmed this by showing a significant recovery of ATP production with co-culture with endometrial organoid-originated mitochondria in TGF-β-induced damaged cells, demonstrating severely decreased ATP production levels compared with normal cells (**Figure 5H, 5L and S5**). Furthermore, restored levels of Hk2 supported mitochondrial transfer-mediated metabolic recovery, accompanied by a remarkable decrease in fibrotic signals (**Figure 5H-K**). In addition, the sole transplantation of mitochondria isolated from endometrial organoids to the AS endometrium showed anti-fibrotic effects and metabolic recovery comparable to those induced by endometrial organoids transplantation (**Figure 5M-O**). Although the specific mode and stimuli of mitochondrial migration to damaged sites, including tunnelling nanotubes, extracellular vesicles, and gap junction channels, are still needed to be elucidated 40, 66, 74, 75, this might indicate that mitochondria transferred from transplanted endometrial organoids are essential for regulating the metabolic milieu for the maintenance of uterine function and fertility 70.

## Conclusions

Significantly, we demonstrated that transplantation of endometrial tissue-derived organoids recovered the damaged structure and impaired function of the AS endometrium by dramatically reducing fibrotic lesions and increasing cellular proliferation and vessel formation, eventually resulting in improved embryo implantation. Moreover, we propose that dysfunctional mitochondria in damaged tissues may be a key cellular feature of the AS endometrium, which we found was fully restored by functional mitochondria transferred from transplanted endometrial organoids. The endometrial organoid-originated mitochondria were found to recover excessive collagen accumulation in fibrotic lesions and restore the shift in the uterine metabolic environment to the levels in normal endometrial levels. Further studies addressing the clinical applicability of endometrial organoids may further aid in identifying new therapeutic strategies for infertility in patients with AS.

## Supplementary Material

Supplementary figures and table 1.

## Abbreviations

AS: Asherman's syndrome
D&C: dilatation and curettage
RIF: recurrent implantation failure
RPL: recurrent pregnancy loss
IUGR: intrauterine growth retardation
NF-kB: nuclear factor kappa-light-chain-enhancer of activated B cell
TIMPs: tissue inhibitors of metalloproteinases
ROS: reactive oxygen species
HSG: hysterosalpingography
TVS: transvaginal ultrasound
MT: masson's trichrome
DAB: 3,3′-Diaminobenzidine
mESC: mouse endometrial stromal cell
HUVEC: human umbilical vein endothelial cell
dPCR: digital polymerase chain reaction
MT: mitochondria
α-SMA: alpha-smooth muscle actin
ERα: estrogen receptor alpha
VIM: vimentin
MUC1: mucin 1
h-MITO: human-mitochondria
PDMS: polydimethylsiloxane
ECM: extracellular matrix
TGF-β: transforming growth factor beta
EGF: epidermal growth factor
HGF: hepatocyte growth factor
VEGF: vascular endothelial growth factor
bFGF: basic fibroblast growth factor
NTC: non template control
FCCP: trifluoromethoxy carbonylcyanide phenylhydrazone
TMRE: tetramethyl rhodamine ethyl ester
mNuDNA: mouse nuclear DNA
mMtDNA: mitochondrial DNA
GFP: green fluorescent protein
EGFP: enhanced green fluorescent protein
TOMM20: translocase of outer mitochondrial membrane 20
NT: Non-transplanted
TP: Transplanted
