# Supplementary material for: Endometrial organoids: a reservoir of functional mitochondria for uterine repair
Source: Theranostics. 2024 Jan 1;14(3):954–72. doi: 10.7150/thno.90538 (PMC10797286; doi:10.7150/thno.90538)
Supplement: Supplementary file 1 — Supplementary figures and table 1. [file thnov14p0954s1.pdf]

Endometrial organoids: a reservoir of functional mitochondria for uterine repair

Sun-Young Hwang[1,#], Danbi Lee[1,#], Gaeun Lee[1,#], Jungho Ahn[2], Yu-Gyeong Lee[1], Hwa Seon Koo[3], Youn-Jung Kang[1,2,*]

Affiliations

1. Department of Biomedical Science, School of Life Science, CHA University; 335 Pangyo-ro, Bundang-gu, Seongnam-si, Gyeonggi-do, South Korea
2. Department of Biochemistry, Research Institute for Basic Medical Science, School of Medicine, CHA University; 335 Pangyo-ro, Bundang-gu, Seongnam-si, Gyeonggi-do, South Korea
3. CHA Fertility Center Bundang; 59, Yatap-ro, Bundang-gu, Seongnam-si, Gyeonggi-do, South Korea

[#]These authors contributed equally to this work

*Corresponding Author: Youn-Jung Kang (yjkang@cha.ac.kr)

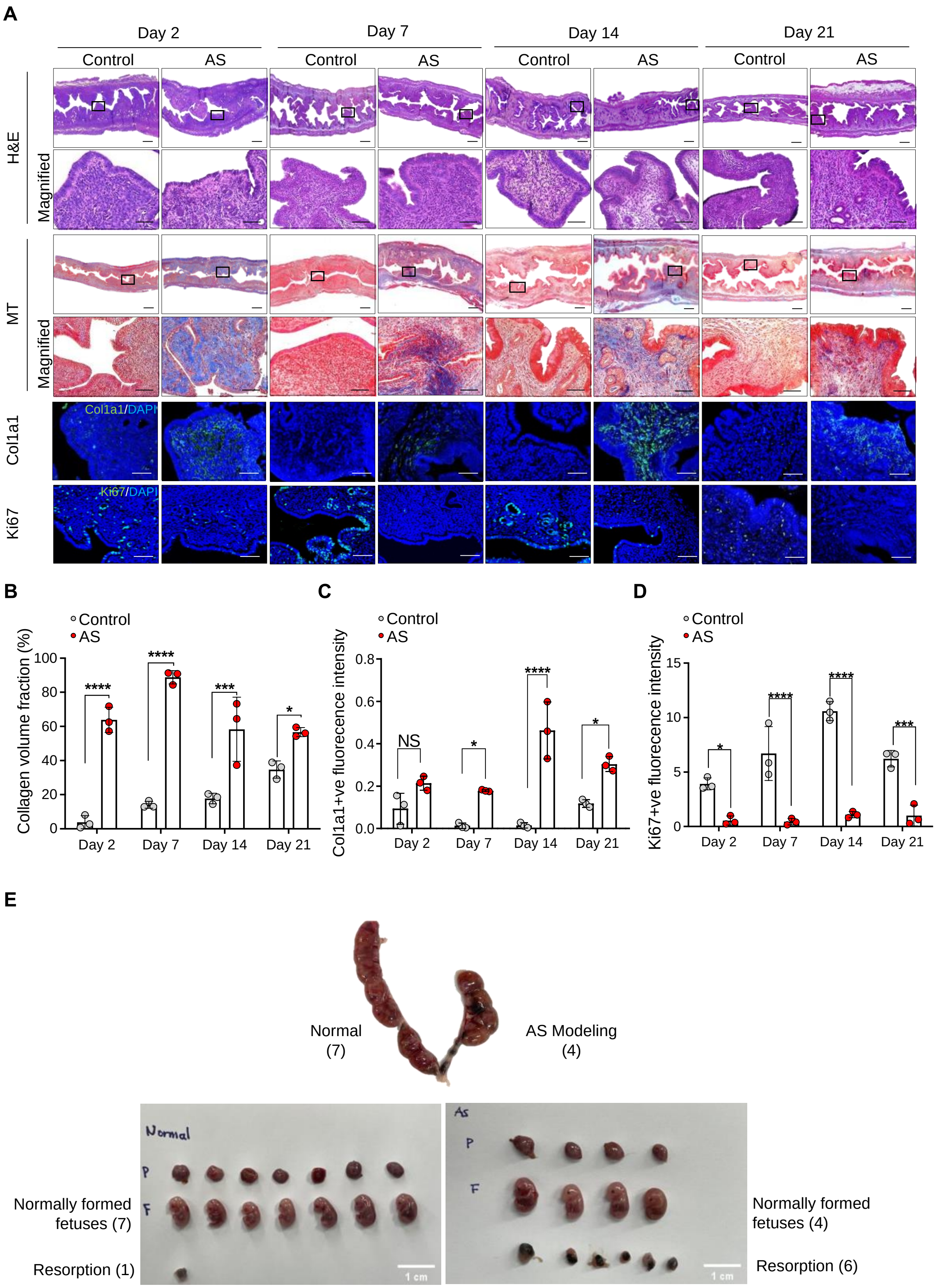
A
Day 2
Day 7
Day 14
Day 21
Control
AS
H&E
Magnified
MT
Magnified
Col1a1
Col1a1/DAPI
Ki67
Ki67/DAPI
B
Control
AS
Collagen volume fraction (%)
100
80
60
40
20
0
****
****
***
*
Day 2
Day 7
Day 14
Day 21
C
Control
AS
Col1a1+ve fluorescence intensity
0.8
0.6
0.4
0.2
0.0
NS
*
****
*
Day 2
Day 7
Day 14
Day 21
D
Control
AS
Ki67+ve fluorescence intensity
15
10
5
0
*
****
****
***
Day 2
Day 7
Day 14
Day 21
E
Normal
(7)
AS Modeling
(4)
Normal
P
F
AS
P
F
Normally formed
fetuses (7)
Resorption (1)
Normally formed
fetuses (4)
Resorption (6)
1 cm
1 cm

Supplementary Figure 1. Successful generation of a murine model of AS

(A) Representative immunostaining images of H&E, MT, Col1a1 (green), and Ki67 (green) and DAPI (blue) of normal and AS-induced endometrium at indicated days (Day 7, 14 and 21). Scale bar; 25um. Collagen volume fraction **(B)**, Col1a1 intensity **(C)**, or $Ki67^+$ fluorescence intensity **(D)** shown in **(A)** was quantified (Total number of mice=48; 3 mice per group; triplicates). Data were expressed as mean ± SD, analyzed using the ordinary two-way ANOVA with Turkey's multiple comparisons test including P-values (*<0.05, **<0.01, ***<0.001, ****<0.0001, NS; not significant). **(E)** Representative images of fetus and placenta from normal and AS-induced horn at pregnancy day 14. Scale bar; 1cm.

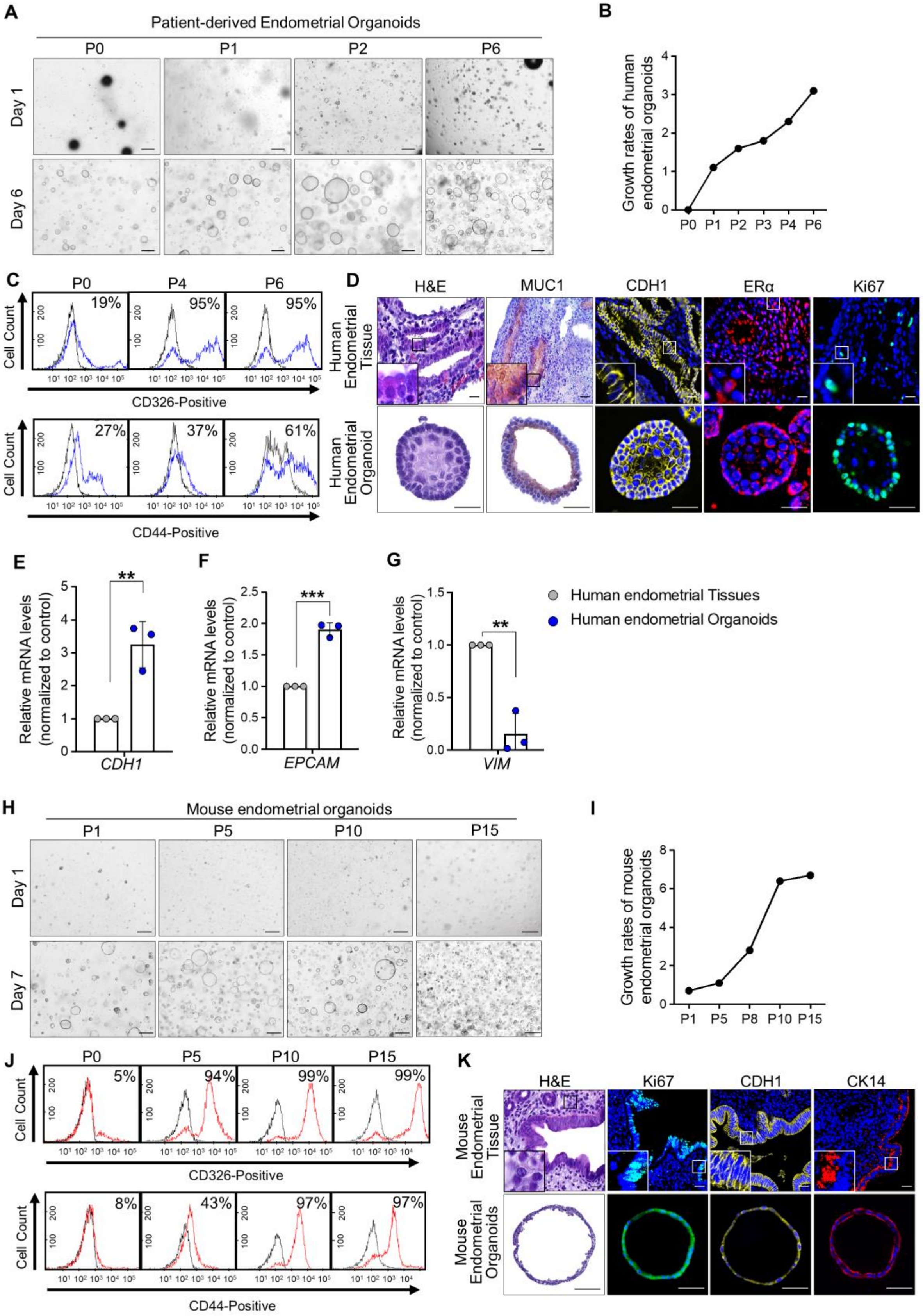
A
Patient-derived Endometrial Organoids
P0
P1
P2
P6
Day 1
Day 6
B
Growth rates of human endometrial organoids
P0 P1 P2 P3 P4 P6
C
P0
P4
P6
19%
95%
95%
Cell Count
CD326-Positive
27%
37%
61%
CD44-Positive
D
H&E
MUC1
CDH1
ERα
Ki67
Human Endometrial Tissue
Human Endometrial Organoid
E
F
G
Relative mRNA levels (normalized to control)
CDH1
EPCAM
VIM
Human endometrial Tissues
Human endometrial Organoids
H
Mouse endometrial organoids
P1
P5
P10
P15
Day 1
Day 7
I
Growth rates of mouse endometrial organoids
P1 P5 P8 P10 P15
J
P0
P5
P10
P15
5%
94%
99%
99%
Cell Count
CD326-Positive
8%
43%
97%
97%
CD44-Positive
K
H&E
Ki67
CDH1
CK14
Mouse Endometrial Tissue
Mouse Endometrial Organoids

46

Supplementary Figure 2. Generation of human or mouse endometrial tissue-derived organoids

(A) Expansion and morphological observation of human endometrial organoids from each passage at day 1 and 6. Scale bar; 25um. **(B)** Growth curve of human endometrial organoids up to passage 6. **(C)** Flow cytometry analyses of CD326 and CD44 (blue) versus isotype controls (grey) in human endometrial organoids from each passage. **(D)** Immunostaining images of H&E, MUC1, CDH1, ERα, and Ki67 in human endometrial organoids and its parental tissues. Scale bar; Tissue-100um (H&E, MUC1), 20um (CDH1, ERα and Ki67), Organoid-100um (H&E, MUC1), 30um (CDH1, ERα and Ki67). QRT-PCR analyses of epithelial markers (*CDH1* **(E)** and *EPCAM* **(F)**) and stromal marker (*VIM* **(G)**) in human endometrial organoids compared with its parental tissues (3 human endometrial organoids were used in characterization). Data were expressed as mean ± SD, analyzed using the unpaired t test including P-values (*<0.05, **<0.01, ***<0.001, ****<0.0001, NS; not significant). **(H)** Expansion and morphological observation of mouse endometrial organoids from each passage at day 1 and 7. Scale bar; 25um. **(I)** Growth curve of mouse endometrial organoids up to passage 15. **(J)** Flow cytometry analyses of CD326 (red) and CD44 (red) versus isotype controls (grey) in mouse endometrial organoids. **(K)** Immunostaining images of H&E, Ki67, CDH1, and CK14 in mouse endometrial organoids and its parental tissues (Four independent sets of human endometrial organoids were used for characterization). Scale bar; Tissue-100um (H&E), 20um (Ki67, CDH1 and CK14), Organoid-100um (H&E), 30um (Ki67, CDH1 and CK14).

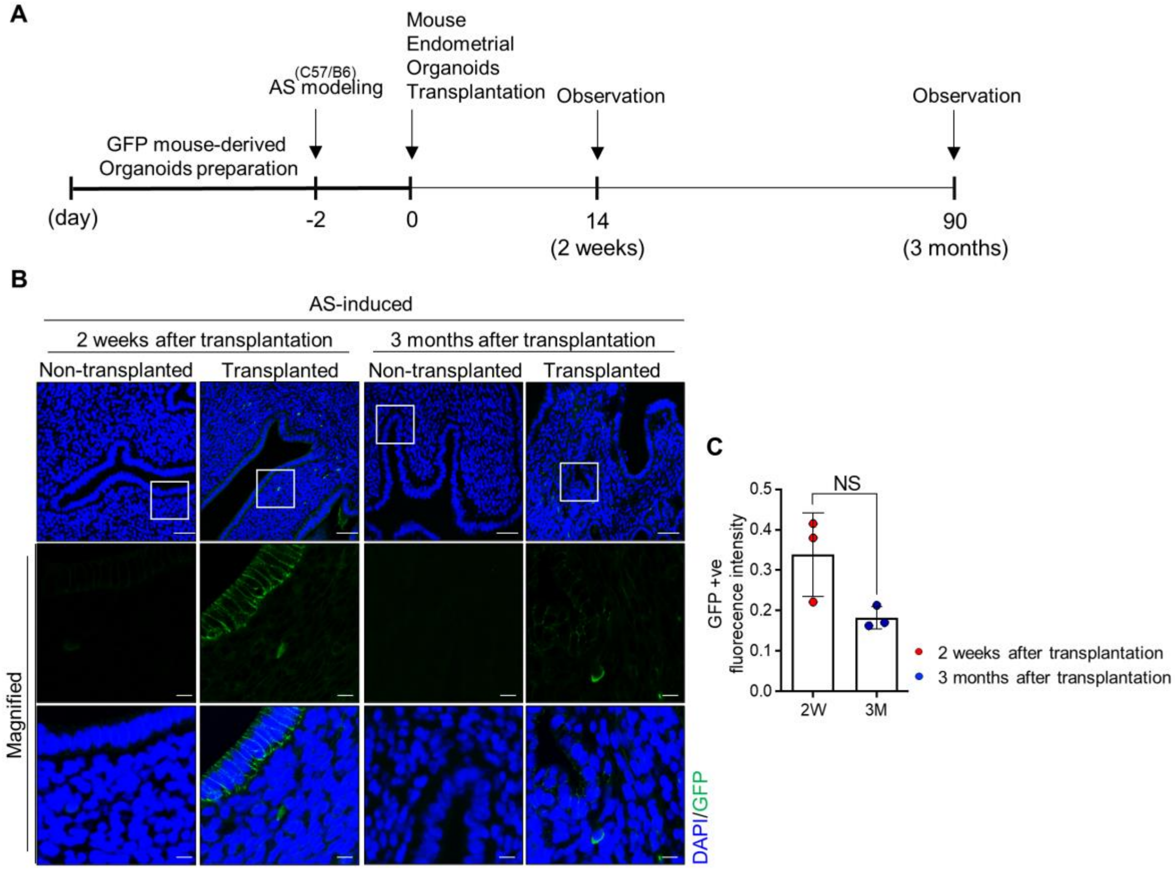

Supplementary Figure 3. Lasting duration time of mouse endometrial organoid transplantation in AS-induced endometrium

(A) An experimental design of GFP-mouse endometrial organoid transplantation to AS-induced uteri of C57/B6 mice. **(B)** Immunofluorescent analyses of GFP (green) and DAPI (blue) in GFP-mouse endometrial organoid-transplanted AS-induced endometrium 3 months after transplantation compared with 2-week time point or non-transplanted AS-induced endometrium (Total number of mice=6; 3 mice per group). Scale bar; 50um, 10um (Magnified). Quantified intensity of GFP signals detected in AS-induced endometrium 3 months or 2 weeks after transplantation of GFP mouse-derived endometrial organoids was shown in a graph **(C).** Data were expressed as mean ± SD, analyzed using the unpaired t test including P-values (NS; not significant).

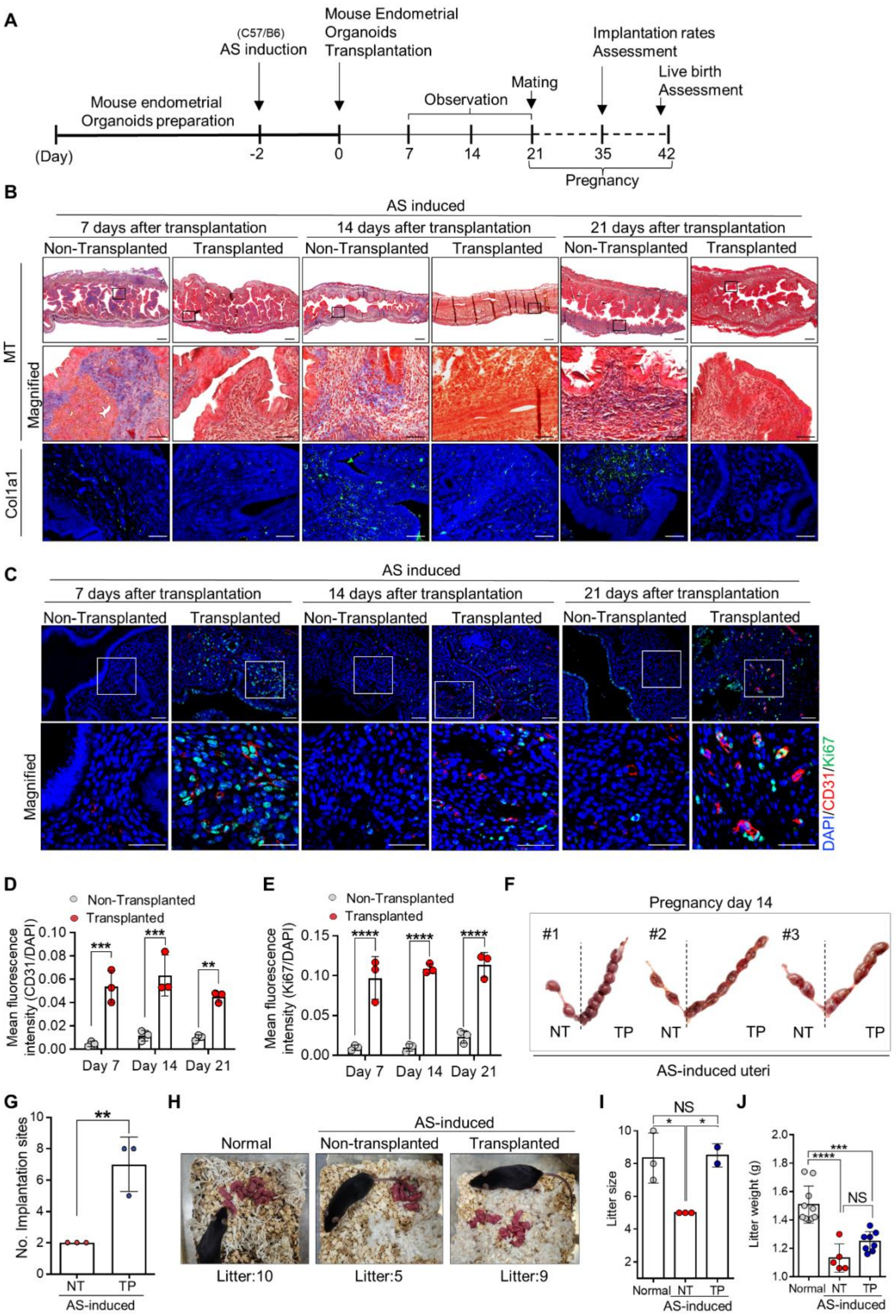
A
(C57/B6)
AS induction
Mouse Endometrial
Organoids
Transplantation
Implantation rates
Assessment
Live birth
Assessment
Mating
Observation
Mouse endometrial
Organoids preparation
(Day)
-2
0
7
14
21
35
42
Pregnancy
B
AS induced
7 days after transplantation
14 days after transplantation
21 days after transplantation
Non-Transplanted
Transplanted
MT
Magnified
Col1a1
C
AS induced
7 days after transplantation
14 days after transplantation
21 days after transplantation
Non-Transplanted
Transplanted
Magnified
DAPI/CD31/Ki67
D
Non-Transplanted
Transplanted
Mean fluorescence intensity (CD31/DAPI)
Day 7
Day 14
Day 21
E
Non-Transplanted
Transplanted
Mean fluorescence intensity (Ki67/DAPI)
Day 7
Day 14
Day 21
F
Pregnancy day 14
#1
#2
#3
NT
TP
AS-induced uteri
G
No. Implantation sites
NT
TP
AS-induced
H
AS-induced
Normal
Non-transplanted
Transplanted
Litter:10
Litter:5
Litter:9
I
NS
Litter size
Normal
NT
TP
AS-induced
J
Litter weight (g)
NS
Normal
NT
TP
AS-induced

Supplementary Figure 4. Therapeutic effects of mouse endometrial organoid transplantation in AS-induced endometrium

(A) An experimental design of mouse endometrial organoid transplantation to AS-induced uteri of C57/B6 mice and further analyses including fertility assessments. **(B)** Immunohistochemical analyses of MT and Col1a1 in mouse endometrial organoid-transplanted AS-induced endometrium compared with non-transplanted AS-induced endometrium at indicated days (Day 7, 14 and 21) (Total number of mice=48; 3 mice per group; triplicates and three independent sets of mouse organoids were used for each transplantation). Scale bar; 25um. **(C)** Immunofluorescent analyses of CD31 (red) and Ki67 (green) with DAPI (blue) in in mouse endometrial organoid-transplanted AS-induced endometrium compared with non-transplanted AS-induced endometrium at indicated days (Day 7, 14 and 21). Scale bar; 25um**.** Immunofluorescent CD31 and Ki67 intensity were quantified in graphs of **(D-E)**. **(F)** Representative images of uteri with implantation sites on pregnancy day 14 (n=3). **(G)** The total number of implantation sites was counted in each horn and quantified. **(H)** Representative images of mouse litters with their mother with endometrial organoids-transplanted AS-induced endometrium compared to groups with non-transplanted AS-induced endometrium and normal endometrium. Comparisons of sizes **(I)** and weights **(J)** of litters from mice with endometrial organoids-transplanted AS-induced endometrium compared to groups with non-transplanted AS-induced endometrium and normal endometrium. Data were expressed as mean ± SD, analyzed using the unpaired t test including P-values (*<0.05, **<0.01, ***<0.001, ****<0.0001, NS; not significant).

A

B

C

D

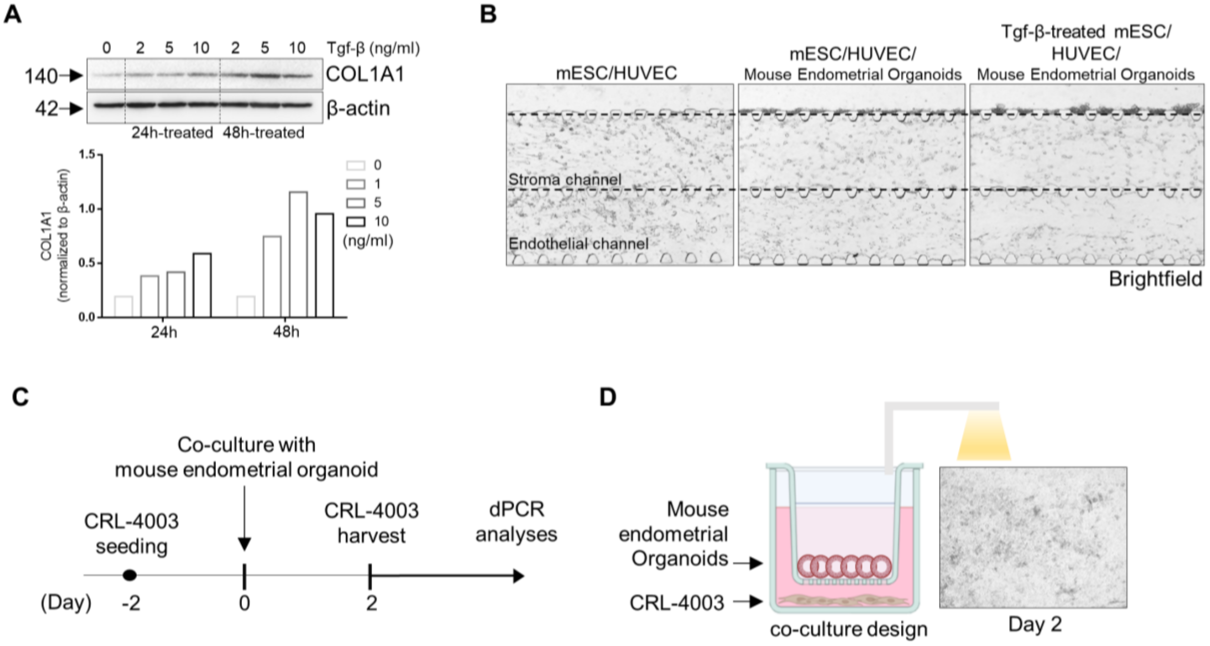

E

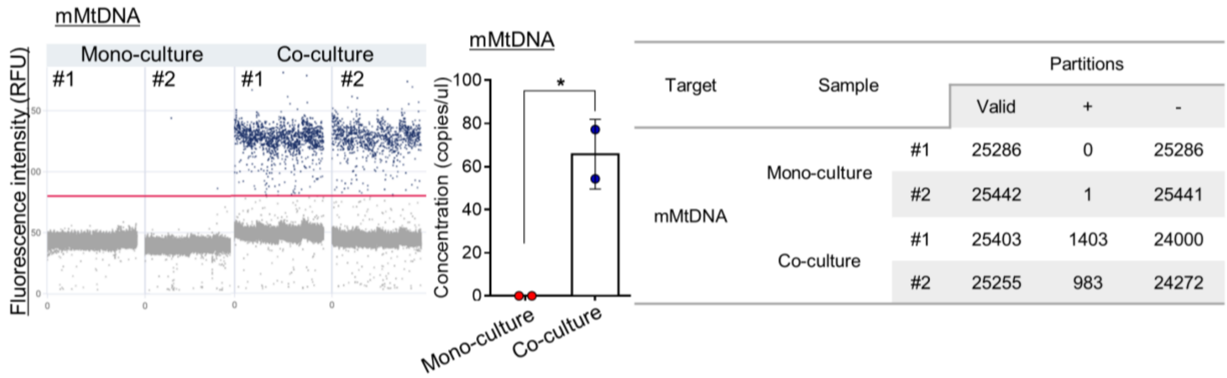

F

Target	Sample		Partitions		
			Valid	+	-
mMtDNA	Mono-culture	#1	25286	0	25286
		#2	25442	1	25441
	Co-culture	#1	25403	1403	24000
		#2	25255	983	24272

G

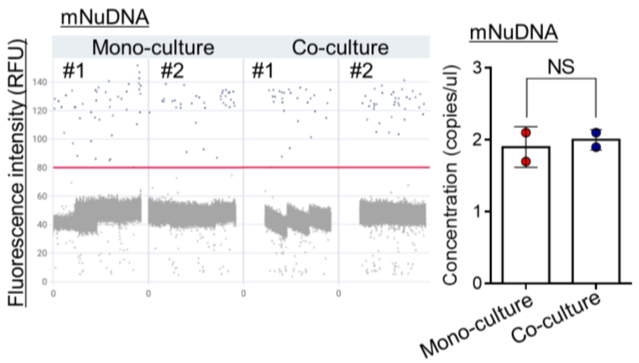

H

Target	Sample		Partitions		
			Valid	+	-
mNuDNA	Mono-culture	#1	25441	45	25396
		#2	25491	35	25456
	Co-culture	#1	25476	39	25437
		#2	25476	42	25434

Supplementary Figure 5. Endometrial organoid-originated mitochondrial migration into Tgf-β-induced damaged cells

(A) Immunoblotting analysis of COL1A1 in mESCs with Tgf-β treatment at indicated concentration (0, 2, 5, and 10 ng/ml) and timepoints (0, 24, and 48 h). **(B)** Brightfield images about serial steps of cell loading for establishment of the endometrium-on-a-chip. Scale bar; 25um. **(C)** An experimental design of co-culture of CRL-4003 with mouse endometrial organoids. **(D)** An experimental design of transwell assay and brightfield image of co-cultured CRL-4003 with mouse endometrial organoids at day 2. **(E-F)** digital PCR analyses of mMtDNA (mouse mitochondrial DNA) and partitioning information in co-cultured CRL-4003 with mouse endometrial organoids compared with monocultured CRL-4003. **(G-H)** dPCR digital PCR analyses of mNuDNA (mouse nucleus DNA) and partitioning information in co-cultured CRL-4003 with mouse endometrial organoids compared with monocultured CRL-4003. Data were expressed as mean ± SD, analyzed using unpaired t test including P-values (*<0.05, **<0.01, ***<0.001, ****<0.0001, NS; not significant).

Supplementary Table 1. Primer sequence pairs used for QRT-PCR and RT-DPCR.

Species	Gene	Direction	Sequence
Mouse	*Rpl7*	Forward	TCAATGGAGTAAGCCCAAAG
		Reverse	CAAGAGACCGAGCAATCAAG
	Actb	Forward	GTGACGTTGACATCCGTAAAGA
		Reverse	GCCGGACTCATCGTACTCC
	Tgfb1	Forward	GTGAAACGGAAGCGCATCGAAG
		Reverse	CATAGTAGTCCGCTTCGGGCTCC
	Timp1	Forward	CTTGGTTCCCTGGCGTACTC
		Reverse	ACCTGATCCGTCCACAAACAG
	Col1a1	Forward	CTGGCGGTTCAGGTCCAAT
		Reverse	TTCCAGGCAATCCACGAGC
	Pgc1a	Forward	ACGGTTTACATGAACACAGCTGC
		Reverse	CTTGTTCGTTCTGTTCAGGTGC
	Nrf1	Forward	GAACGCCACCGATTTCACTGTC
		Reverse	CCCTACCACCCACGAATCTGG
	Mfn1	Forward	TGCATGTTTCACCACAGTTTC
		Reverse	GTAGCTCACAACCACCTGTAA
	Fis1	Forward	AGGCTCTAAAGTATGTGCGAGG
		Reverse	GGCCTTATCAATCAGGCGTTC
	mMtDNA	Forward	AGGCATGAAAGGACAGCACA
		Reverse	TTGGGGTTTGGCATTAAGAGGA
	mNuDNA	Forward	GAATTCAGATTTGTGCATACACAGTGACT
		Reverse	AACATTTTTCGGGGAATAAAAGTTGAGT
	Tnfa	Forward	CCCTCACACTCAGATCATCTTCT
		Reverse	GCTACGACGTGGGCTACAG
	Hk2	Forward	GGAGGAACCAATTTCCCTGCTGCT
		Reverse	CCTTTGATCCCCATGTATCCAAGA
	Scd1	Forward	TTCTTGCGATACACTCTGGTGC
		Reverse	CGGGATTGAATGTTCTTGTCGT
	Gpr84	Forward	CTCCTGCTACCATGAGTCTGT
		Reverse	GTGCAGTAGAGTAGATCAGCCA
Human	*ACTB*	Forward	CATGTACGTTGCTATCCAGGC
		Reverse	GCCTTAATGTCACGCACGAT
	CDH1	Forward	AGTCACTGACACCAACGATAAT

	Reverse	ATCGTTGTTCACTGGATTTGTG
EPCAM	Forward	GTCTGTGAAAACTACAAGCTGG
	Reverse	CAGTATTTTGTGCACCAACTGA
VIM	Forward	CGGCTGCGAGAGAAATTGC
	Reverse	CCACTTTCCGTTCAAGGTCAAG
ITGB3	Forward	AGTAACCTGCGGATTGGCTTC
	Reverse	GTCACCTGGTCAGTTAGCGT
SPP1	Forward	GAAGTTTCGCAGACCTGACAT
	Reverse	GTATGCACCATTCAACTCCTCG
